# A cost-effectiveness analysis of pre-pregnancy genetic screening for deafness: an empirical study in China

**DOI:** 10.3389/fpubh.2023.1081339

**Published:** 2023-12-07

**Authors:** Yipeng Lv, Zhili Wang, Ling Yuan, Fan Cheng, Hao Wu, Zhaoxin Wang, Tao Yang, Ying Chen

**Affiliations:** ^1^School of Public Health, Shanghai Jiao Tong University School of Medicine, Shanghai, China; ^2^Department of Otolaryngology-Head and Neck Surgery, Shanghai Ninth People’s Hospital, Shanghai Jiao Tong University School of Medicine, Shanghai, China; ^3^Ear Institute, Shanghai Jiao Tong University School of Medicine, Shanghai, China; ^4^Shanghai Key Laboratory of Translational Medicine on Ear and Nose Diseases, Shanghai, China; ^5^Department of Endodontics, Stomatological Hospital and Dental School of Tongji University, Shanghai Engineering Research Center of Tooth Restoration and Regeneration, Shanghai, China; ^6^The First Affiliated Hospital, Hainan Medical University, Haikou, Hainan, China

**Keywords:** pre-pregnancy genetic screening, deafness screening, cost-effectiveness, cost utility analysis, public health intervention, health in China

## Abstract

**Objectives:**

This research aims to assess the effectiveness and cost-effectiveness of pre-pregnancy deafness screening policies.

**Methods:**

Married couples from Shanghai, Beijing, and Suzhou in China were enrolled. We conducted high-throughput, pre-pregnancy genetic screenings for deafness in women and their partners. We compared the cost-effectiveness of deafness genetic screening with the status quo. The two-step screening (wife then partner) and following treatments and interventions were included in the decision tree model. We conducted a cost-effectiveness analysis based on the decrease in deaf newborns, healthy newborn births, and cost-utility analysis of pre-pregnancy deafness genetic screening separately. Cost, utility, and probability data used in the three models were collected from a survey combined with literature and expert consultants. A 5% discount rate and a series of one-way sensitivity analyses along with a Monte Carlo simulation were used to test the reliability of this research.

**Results:**

Between Jan 1, 2019, and Dec 31, 2021, we recruited 6,200 females and 540 male spouses from community health service centers in Shanghai, Beijing, and Suzhou. The incremental cost-effectiveness ratio (ICER) for reducing deaf newborn births was USD 32,656 per case and USD 1,203,926 per case for increasing one healthy newborn birth. This gap exists because of the overall decrease of newborn births. From the perspective of the whole society, deafness genetic screening is not cost-effective for reducing the overall quality-adjusted life years (QALY) in the population.

**Discussion:**

Pre-pregnancy genetic testing is effective in decreasing the occurrence of congenital deafness. It is a cost-saving measure when compared with the costs of future medical expenditure and income loss for the affected families. However, such screening and preventive avoidance of pregnancy will decrease the population size and QALY. Only post-screening ART with PGT was shown to increase the birth of healthy newborns. Focusing on key groups such as premature births or consanguineous couples may improve the societal effects of screening.

## Introduction

Congenital deafness has many effects on the quality life of the affected individual and their family and impacts their society. Without timely diagnosis and treatment, deafness can impair language acquisition, mental health, education, work, and income opportunities. The incidence of congenital deafness is 1–3% worldwide; over 30,000 newborn cases are identified in China every year (1, 2). Approximately 50% of congenital deafness is hereditary (3). Congenital deafness can impair quality of life and reduce quality-adjusted life-years (QALY), increasing the societal burdens of disease. Cochlear implants (CI) and hearing aids (HA) in conjunction with speech therapy can help improve the quality of life and communication skills in hearing-impaired children (4). However, these interventions are not curative and may not completely return the affected individual’s quality of life to normal levels.

Genetic factors are responsible for over 50% of hearing loss encountered in neonates and nearly 40% in children (5, 6). Approximately 80% of genetic hearing loss is autosomal recessive; many cases are born from spouses without a family history of congenital or childhood hearing loss. Among these cases, mutations in the deafness genes *GJB2* and *SLC26A4* are the most prevalent in many countries, including China (PMID: 31564438, 30890784).

The main intervention approaches for preventing congenital deafness involve three strategies. Primary prevention involves deafness genetic screening, genetic counseling, and fertility guidance before pregnancy. Secondary prevention is prenatal deafness genetic screening and diagnosis. Tertiary prevention is newborn hearing screening, diagnosis, and intervention with language rehabilitation. Pre-pregnancy deafness genetic screening can identify risks for deafness and allow parents to make informed pregnancy-related choices around their risk of birth to children with hearing loss. Genetic diagnosis of hearing loss can help to avoid unnecessary and costly clinical testing, offer prognostic information, and guide future medical management. The importance of an etiological diagnosis is underlined by the 2014 American College of Medical Genetics and Genomics (ACMG) guidelines for the diagnosis of hearing loss, which recommended that genetic testing should be included in the workup of patients with non-syndromic hearing loss (NSHL) (7).

Currently, the universal newborn hearing screening program (UNHS) has been widely used as a hearing screening program in many countries around the world with otoacoustic emission (OAEs) and automated auditory brain stem response (AABR) technologies (2). Conversely, pre-pregnancy genetic screening strategies, as part of a hearing loss prevention policy, remain underutilized in most countries (7). Next-generation sequencing (NGS) technology has been widely implemented in the genetic diagnosis of hearing loss. However, relatively few countries utilize this technology as part of a national policy for pre-pregnancy deafness screening. Given the limits of health funding, understanding the cost-effectiveness of such a policy in China is critical. Therefore, in this study, we collected cost and effectiveness data to assess the effect, utility, and cost-effectiveness of pre-pregnancy deafness screening policy from the perspective of society and affected families. As the prevention of disability and promotion of a healthy population are also important goals for policymakers, the cost-effectiveness for the overall population was also examined.

## Methods

### Study design

In this study, we performed a high-throughput, pre-pregnancy genetic screening for deafness in women and their spouses from the general population. We used targeted NGS that covers 45 common mutations in the *GJB2* and *SLC26A4* genes (Supplementary Table S1). We collected information about the epidemiological characteristics associated with these deafness-related genes and conducted a cost-effectiveness analysis with expected reproductive outcomes and corresponding costs. We used a two-step screening strategy. In the first step, women planning pregnancy received genetic screening; if negative, their involvement in the study was marked as complete. If pathogenic recessive mutations in the deafness-related genes were identified, their partners were screened in the second step. Based on the results of the genetic screenings of the couples, families were divided into four different risk categories including high-risk, medium-high risk, medium-low risk, and low-risk which reflected their odds of delivering a newborn with genetic hearing loss. These risk categories and their following treatments after genetic screening were shown as follows:

(1) High-risk families: both husband and wife have biallelic recessive mutations in the same gene, and the newborn is very likely to have genetic deafness. For these families, only pre-pregnancy medical counseling services were provided regarding the likely hearing loss. The couples were free to decide whether or not to give birth based on this information.
(2) Medium-high risk families: one of the spouses has biallelic recessive mutations and the other has a single heterozygous recessive mutation in the same gene. These couples can expect a 50% chance of delivering a child with genetic deafness.
(3) Medium-low risk families: Both spouses have a single heterozygous recessive mutation in the same gene. There is an approximately 25% chance of delivering a child with genetic deafness.
(4) Low-risk families: the woman’s pregnancy genetic screening result is negative. The chance of delivering a child with genetic deafness is relatively low.

For medium-risk families (categories 2 and 3) the main follow-up interventions included: (1) choosing not to have children; (2) normal pregnancy; (3) normal pregnancy with a prenatal amniocentesis (if amniocentesis screening was positive, couples could decide whether or not to terminate the pregnancy according to the local legal and ethical regulations); (4) utilize assisted reproductive technologies (ART) with a preimplantation genetic test (PGT) and proceed to implantation of fertilized eggs with the desired genotypes.

### Subjects and public involvement

**Figure 1:**
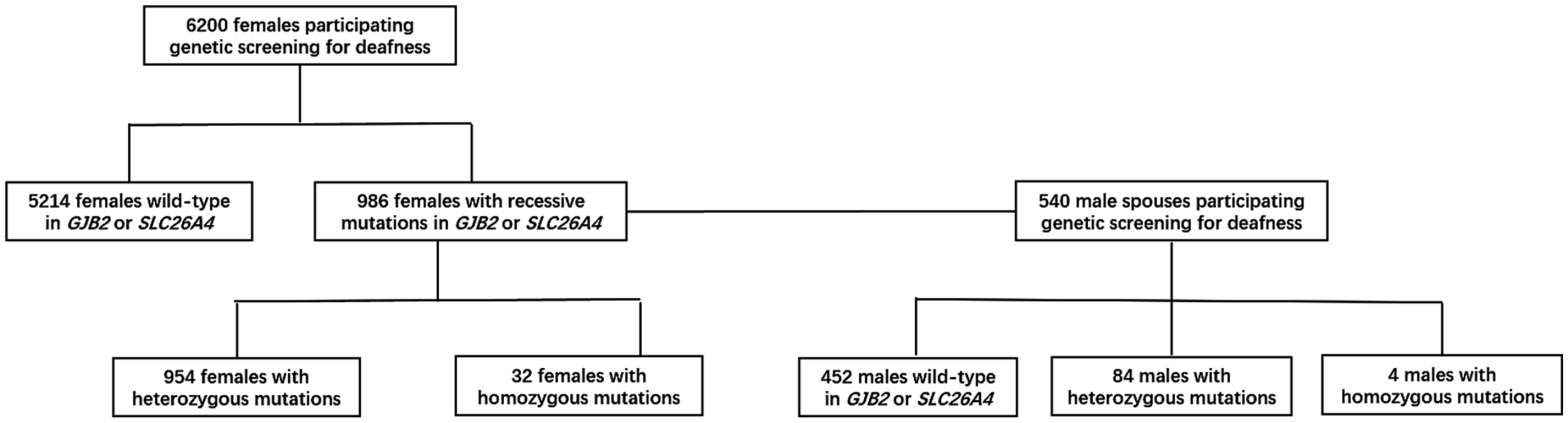
The flowchart of objects participating the research and their genetic screening results.

From 2019 to 2021, 6,200 females and 540 male spouses from the general population were recruited from community health service centers in Shanghai, Beijing, and Suzhou (Figure 1); this study involved research teams from the Shanghai Ninth People’s Hospital, the Haidian District Maternal and Child Health Care Hospital, and the Suzhou Science and Technology Town Hospital, respectively. The inclusion criterion for female participants was being aged between 20 and 55 years. The sole exclusion criterion was an inability to provide complete demographic and health information as required by this study. All participants provided informed consent before participation and the study was conducted per approval by the Ethics Committee of Ninth People’s Hospital, Shanghai Jiaotong University School of Medicine.

Genomic DNA was extracted from blood samples provided by the participants. Sequences covering the 45 common mutations for deafness (Supplementary Table S1) were captured by a customized capture assay (Fujun Genetics, Shanghai, China) and sequenced on an Illumina NovaSeq 6000-PE150 platform (Illumina, San Diego, CA, USA). Pathogenic mutations were confirmed by Sanger sequencing.

### Model generation

**Figure 2:**
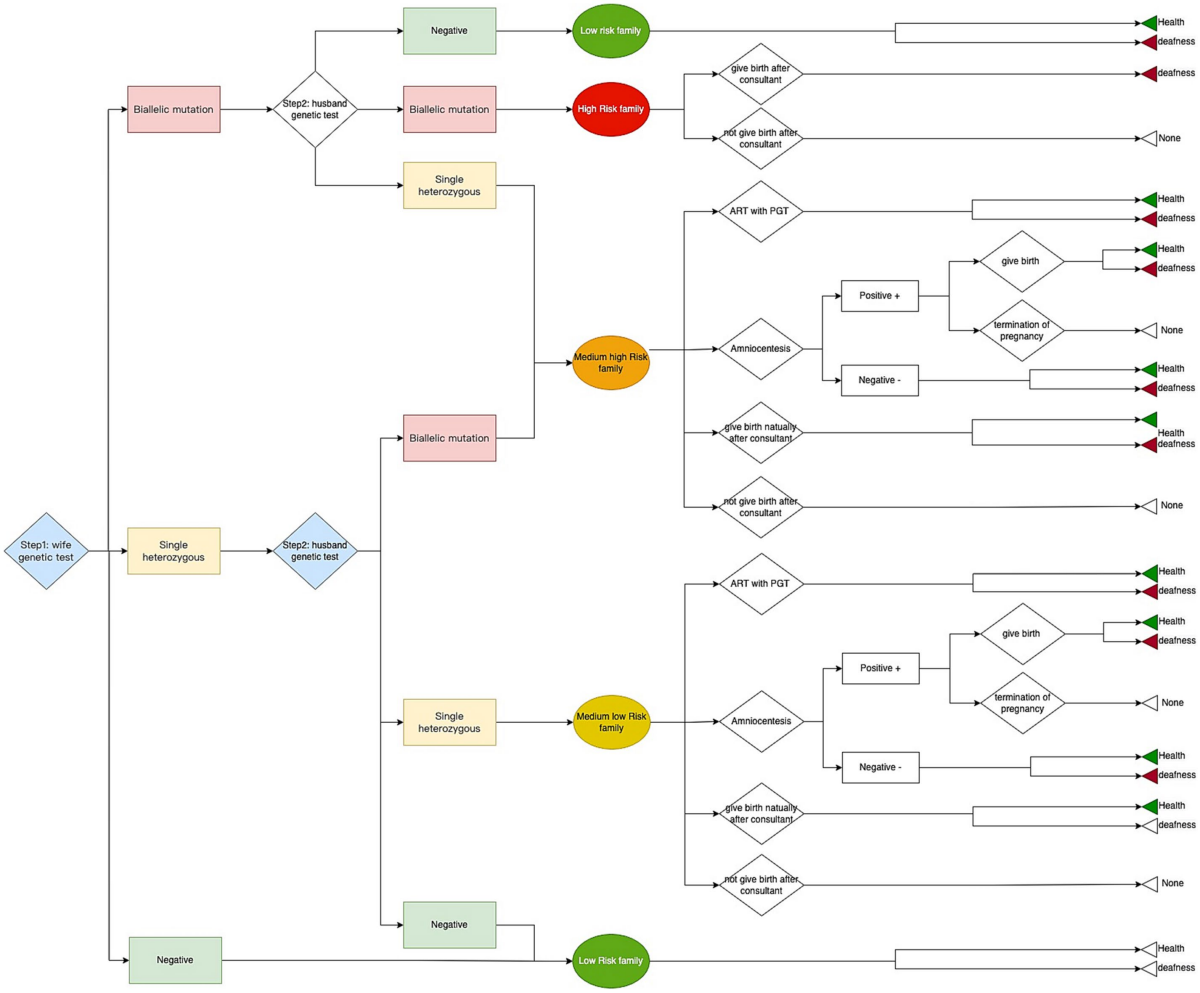
Schematic diagram of the decision tree for deafness screening.

The genetic deafness screening was conducted in two steps as previously mentioned. The corresponding treatment and intervention were carried out based on the test result and the couple’s decision. A decision tree model was used in the research and we compared the deafness genetic screening strategy with the status quo, which in essence means the absence of any intervention that alters the course of the pregnancy. All possible options available to couples following screening were included in the decision tree (mentioned in the STUDY DESIGN part). The three possible outcomes were the birth of a healthy newborn or a deaf newborn and none (Figure 2). All data such as costs, utilities, the probability of different arms in the model were collected from the survey combined with literature and an expert consultant (Tables 1, 2). We analyzed the cost, effect, and ICER between the screening strategy and status quo. The model was calculated by TreeAge Pro (Healthcare Version, 2022 version; TreeAge Software, Inc., Williamstown, MA).

**Table 1:** Cost of pre-pregnancy deafness genetic screening and subsequent interventions (2021, USD).

Items	Value	Source
Direct cost ($/case)		
Deafness genetic screening cost	52.20	Collected from survey results
Labor cost for screening	9.00	
Screening project promotion cost	3.00	
Reproductive cost^*^	651.00	(8, 9)
ART with PGT	11,940.30	Collected from survey results
Amniocentesis	2,686.60	
Indirect cost ($/case)		
Productivity loss of ART with PGT	28.70	(10, 11)
Productivity loss of Amniocentesis	14.40	
Future cost ($/life)^†^		
Future income of healthy individuals	115,622.00	(11, 12)
Future income of disabled individuals	65,996.00	
Future medical expenditure of health individuals	6,961.40	
Future medical expenditure of disabled individuals	11,142.40	

The average hourly wage in urban and rural China is $3.19 based on the 2021 National Economic and Social Development Statistical Bulletin and the 2018 National Time Use Survey Bulletin issued by the National Bureau of Statistics. Productivity loss of ART and Amniocentesis was 1 and 0.5 days, respectively.

* The reproductive cost was calculated as the cost of natural and cesarean births with their respective proportions in China.

† Productivity loss was calculated according to the utilized days.

**Table 2:** The probability of each arm used in the model.

No.	Items	%	Sources
1	Wife biallelic mutation	0.0050	Actual data and expert advice
2	Wife single heterozygous mutation	0.1572	Actual data
3	Wife passes screening (−)	0.8378	
4	Husband Biallelic Mutation	0.0074	
5	Husband single heterozygous mutation	0.1370	
6	Husband passes screening (−)	0.8556	
7	High-risk families choose to give birth naturally	0.1930	(17)
8	High-risk families choose not to have children	0.8070	
9	Medium-risk families choose to take the ART with PGT	0.1774	(18) Combined with expert advice
10	Medium-risk families choose amniocentesis	0.7096	
11	Medium-risk families choose to give birth naturally	0.0565	
12	Medium-risk families choose not to have children	0.0565	
13	Positive amniocentesis in medium-high-risk (+)	0.5000	Calculated by Mendelian laws of inheritance
14	Negative amniocentesis in medium-high-risk family (−)	0.5000	
15	Positive amniocentesis in medium-low-risk family (+)	0.2500	
16	Negative amniocentesis in medium-low-risk family (−)	0.7500	
17	Medium-risk families give birth with positive amniocentesis (+)	0.1930	(17)
18	Medium-risk families end the pregnancy with positive amniocentesis (+)	0.8070	
19	Low-risk families have healthy newborns	0.9990	(6, 18)
20	Low-risk families have deaf newborns	0.0010	
21	Medium-risk families have healthy newborns after ART with PGT	0.9990	(19)
22	Medium-risk families have deaf newborns after ART with PGT	0.0010	
23	Medium-risk families have healthy newborns with positive amniocentesis (+)	0.0010	Expert advice
24	Medium-risk families have deaf newborns with positive amniocentesis (+)	0.9990	
25	Medium-risk families have healthy newborns with negative amniocentesis (−)	0.9990	(20)
26	Medium-risk families have deaf newborns with negative amniocentesis (−)	0.0010	
27	Medium-high-risk families have healthy newborns naturally	0.5000	
28	Medium high-risk families have deaf newborns naturally	0.5000	
29	Medium low-risk families have healthy newborns naturally	0.7500	Expert advice
30	Medium low-risk families have deaf newborns naturally	0.2500	
31	Low-risk families have healthy newborns naturally	0.9990	(6, 21)
32	Low-risk families have deaf newborns naturally	0.0010	

### Cost analysis

We included direct medical costs, direct non-medical costs, and indirect costs. The direct medical costs mainly included the screening cost and the cost of medical care after screening, which included the genetic screening cost [calculated by the fixed cost of equipment and the variable cost of a single test, like alcohol swabs, kits (cassette and buffer), gloves, biosecurity devices, electricity, and water consumption], screening-related medical staff salaries, ART with PGT costs, amniocentesis costs, and maternity expenses. The total cost of genetic screening for deafness was 350.00RMB per person, based on contracted service for next-generation sequencing provided by Fujun Genetics, Shanghai, China. The direct non-medical costs included the promotion costs of the screening project including advocacy meetings with authorities, training, supervision, and monitoring. Because the deafness gene screening was carried out in community health centers close to the participants’ homes, we did not include costs for additional space or transportation. Indirect costs mainly refer to productivity loss due to the time spent as part of diagnosis, treatment, and medical examination. The cost is calculated based on the *per capita* disposable income of urban and rural areas in China in 2021, as shown in Table 1.

Furthermore, we expected significant differences in future income and medical expenditure between deaf and healthy children. The literature indicates that 47% of the costs arising from deafness are related to the loss of quality of life and 32% are related to additional health and medical expenditure (13). In addition to the provision of hearing aids and cochlear implants, the medical expenses incurred by deaf newborns and their families also include follow-up hearing and speech rehabilitation, as well as future medical costs due to injuries and other services arising from deafness. As a result, the cost of deafness-related medical care is difficult to measure in detail and can vary widely based on individual situations; unfortunately, comprehensive research in this area is lacking. In this study, for both deaf and healthy people have medical expenditure and productivity income. This component was calculated based on the annual *per capita* consumption expenditure of disabled households and national resident households from the “*China 2019 National Survey Report on Income of Disabled Households*,” which are $402.50/year and $251.50/year, respectively. The annual growth rate of health care expenses is calculated by using the 2021 and 2019 annual *per capita* consumption expenditures of households across China. As a result, the estimated 2021 medical expense for disabled individuals is $505.20/year. The total expenditure in life years is calculated utilizing the Chinese life table and a 5% discount rate as suggested by Guidelines for the Evaluation of Chinese Pharmacoeconomics (Table 1).

#### Loss of future income

The future labor loss of deaf individuals includes two components: the loss of earning capacity caused by deafness and disability to the individual and to the family members who care for the deaf individual. This cost was estimated by utilizing the *per capita* disposable income of households with disabilities. According to the “*2019 National Survey Report on Income of Households with Disabilities*,” the *per capita* disposable incomes of Chinese families with disabilities and national residents in 2018 were $2,040.80/year and $4,213.10/year, respectively. Based on the *per capita* disposable income of Chinese residents in 2021 ($5,243.00) from the National Bureau of Statistics of China, the three-year average annual growth rate was calculated to be 7.56% which resulted in the *per capita* disposable income of Chinese families with disabilities in 2021 to be estimated as $2,992.70. The expenses were calculated according to the Chinese life table with a 5% discount rate, as shown in Table 1 (calculation shown in Supplementary appendix Tables S2, S3).

Monetary costs were adjusted to the average 2021 US Dollar exchange value and are listed in Table 1. A 5% discount rate was used based on recommendations of the China Pharmacoeconomics Committee. In this study, all data such as productivity loss, income, medical expenditure, etc. were translated into the 2021 USD value based on a 5% discount rate. We ran the model in TreeAge Pro (TreeAge software, Williamstown, MA, USA).

### Outcome variables and willingness-to-pay

There were two possible pregnancy outcomes: (1) healthy newborns with normal hearing, or (2) newborns with congenital deafness. Due to the complex association between the severity of congenital deafness and the various pathogenic mutations, we did not attempt to subgroup outcomes in this study. The likelihood of outcome was analyzed according to the four family risk subgroup categories.

We built three different models to achieve our research aims: (1) model 1: A cost-effectiveness analysis of taking steps to reduce the birth of deaf newborns. We set the deafness outcome as 1 and the health outcome as 0 and tested the effect of reducing the number of deaf infants. The resulting gap in the model between the screening arm and the status quo arm is the cost-effectiveness of deaf newborns in these two scenarios; (2) model 2: A cost-effectiveness analysis of increasing the birth of healthy newborns. We set the health outcome as 1 and the deafness outcome as 0 and tested the impact of the screening strategy on the birth of healthy newborns; (3) model 3: A cost-utility and policy feasibility analysis of pre-pregnancy deafness genetic screening. The severity of genetic deafness in newborns varies with age. Furthermore, we also built another model to explore the effect of genetic screening on the overall population. This model is not the main focus of this study, so it is shown in Supplementary Appendix. We calculated the health utilization of deaf newborns based on the proportion of different degrees of deafness in China in 2019 from the Chinese global burden of disease data (GBD) multiplied by the utility of different deafness levels from literature, which is 0.91 (14). According to the Chinese life table (15) and the 5% discount rate, the QALY of a healthy person in China is 22.1. Therefore, we set the utility as 22.1 for healthy newborns and 20.1 for deaf newborns (calculation shown in the Supplementary appendix Table S2).

However, the willingness to pay (WTP) range is difficult to translate to the benefit of birth outcomes. There is an evidence gap in fertility-specific WTP guidelines in the field (16). Therefore, for the cost effectiveness analysis (model 1 and 2 to analyze the impact of policy on births of healthy and deaf children), we did not compare it to the regular WTP, but rather compared it to the disease-related opportunity cost. In cost-utility analysis, for the results represented as the QALYs, we compare the ICER for overall QALYs with the whole WTP for the life value. Specifically, China’s *per capita* GDP in 2021 was 12,086.00 USD and 22.1 years for QALY for a healthy person’s whole life. Consequently, we estimate the WTP in this study as 801,302 which is three times the *per capita* GDP in China for the whole QALY (calculation shown in the Supplementary appendix Tables S2, S3).

### Probability of different arms in the model

Table 2 shows the specific values and data sources of the various genotype-intervention pairs and following the various interventions and their probability of corresponding results (the birth of deaf or healthy newborns). The incidence of autosomal recessive pathogenic mutations in the common deafness genes *GJB2* and *SLC26A4* among the Chinese population was collected in the overall cohort of 6,740 individuals. The probability of amniocentesis in the different groups was calculated based on the Mendelian inheritance law. The remaining data were obtained from a literature review combined with expert consultation in the field. For arms with no intervention, the probability of a birth of deaf and healthy newborns was based on Mendelian laws of inheritance.

### Sensitivity analysis

Both one-way deterministic and simulated probabilistic sensitivity analyses were conducted to assess the robustness of the main outcomes. For the one-way sensitivity analysis, a change of plus or minus 10% (for prevalence, utility, sensitivity, specificity, compliance, and transition probability from published literature) and plus or minus 20% of the original values for the parameters were used for probability-related data in sensitivity analysis.

For the probabilistic sensitivity analyses, we set the distribution for cost and transition probability. The cost mainly includes the key costs involved in the deafness screening strategy, including the cost of screening, the cost of PGT, and the cost of amniotic fluid examination. As for the transition probability, since our data are derived from the results of real-life research studies, we do not design the distribution of deafness gene proportion, which is mainly the proportion of (1) high-risk families (couples who are heterozygous and whose children are born to be with congenital deafness) who choose to continue to give birth naturally; (2) medium-risk families (both parents are heterozygous and whose children have a 50% probability of congenital deafness in natural birth) who choose to take the ART with PGT; (3) medium-risk families choose to give birth naturally with amniocentesis; (4) medium-risk families still choose to give birth naturally without any intervention after being informed of the risks; and (5) medium-risk families choose not to have children. These probabilities are susceptible to subjective influences, and can affect cost-effectiveness outcomes to a large extent. By designing the distribution and conducting sensitivity analyses based on these changeable elements, which have strong influence on the outcomes of a deafness screening strategy, it is possible to better understand the conditions under which such a policy would be more effective, and the patterns of change that are influenced by both environmental and individual subjective variables. A β distribution was applied to prevalence, utilities, and transition probabilities, and a γ distribution was applied to cost parameters.

The random error associated with an estimate for the values was included within a plausible range.

## Results

### Study population

Between Jan 1, 2019, and Dec 31, 2021, we recruited 6,200 females and 540 male spouses from community health service centers in Shanghai, Beijing, and Suzhou. For women who tested positive, we were able to obtain all (100%) of the associated male partner samples. The distribution of the detected target genes is shown in Table 3.

**Table 3:** Study population and their deafness genes distribution.

Characteristic	Wild-type *N* (%)	Mono-allelic *N* (%)	Bi-allelic *N* (%)	Total (*N*)
Sex				
Male	452(83.70)	84(15.56)	4(0.74%)	540
Female	5,214(84.1)	954(15.4)	32(0.5)	6,200
Location				
Shanghai	5,141(83.6)	974(15.9)	33(0.5)	6,148
Beijing	446(89.4)	52(10.4)	1(0.2)	499
Suzhou	79(84.9)	12(12.9)	2(2.2)	93

### Measurement of health effects

First, as shown in Table 4, compared with the status quo, each instance of deafness genetic screening was found to reduce the birth of 0.0051 deaf newborns. In other words, 196 families were screened to reduce the birth of deaf newborns by one. Secondly, we looked at the effects on healthy newborns and found that the screening had a small impact on newborns. 7,226 families needed to be screened to increase the birth of healthy newborns by one. Third, our cost-utility analysis showed that genetic screening reduced 0.099 QALY, mainly due to the fewer births of deaf children which resulted in a lower overall QALY.

**Table 4:** ICER of the three different models.

Category	Strategy	Cost	Incr cost	Effectiveness	Incr eff	ICER
Deaf = 1	Status quo	651.00		0.0073		
	Screening	817.60	166.60	0.0022	−0.0051	Dominated^a^
Health = 1	Status quo	651.00		0.9927		
	Screening	817.60	166.60	0.9928	0.0001	1,203,926.40
Utility	Status quo	−107,615.66		22.09		
	Screening	−107,184.25	431.41	21.99	−0.0995	Dominated^b^

The results were negative, indicating that the screening strategy did not improve the QALY of the whole society.

a Dominated (i.e., costs more and less effectiveness) VS. Status Quo. The number of deaf newborn births becomes less under the screening strategy.

b Dominated (i.e., costs more and less effectiveness) VS. Status Quo. In the cost utility analysis, we compared the impact of the screening strategy on QALY with the current situation.

### Cost-effectiveness ratios

In general, two types of costs were included in this study: (1) genetic screening and subsequent medical intervention costs; and (2) future lifetime income and medical expenditure. In part 1 and 2, for the cost-effectiveness analysis of reducing deaf newborns and increasing healthy ones, we included only the costs of screening and the resultant interventions. In part 3, the utility analysis of deafness genetic screening, both the medical expenditures and future income were considered.

As shown in Table 4, the ICER associated with a reduction of deaf newborn births was $32,656.00/case and $1,203,926.00/case for increasing the birth of healthy newborns. From a societal perspective, we found that genetic screening for deafness is not cost-effective for reducing the overall societal QALY. A single screening cost $432.00 and the ICER of utility was $4336.40 /QALY.

### Sensitivity analysis

**Figure 3:**
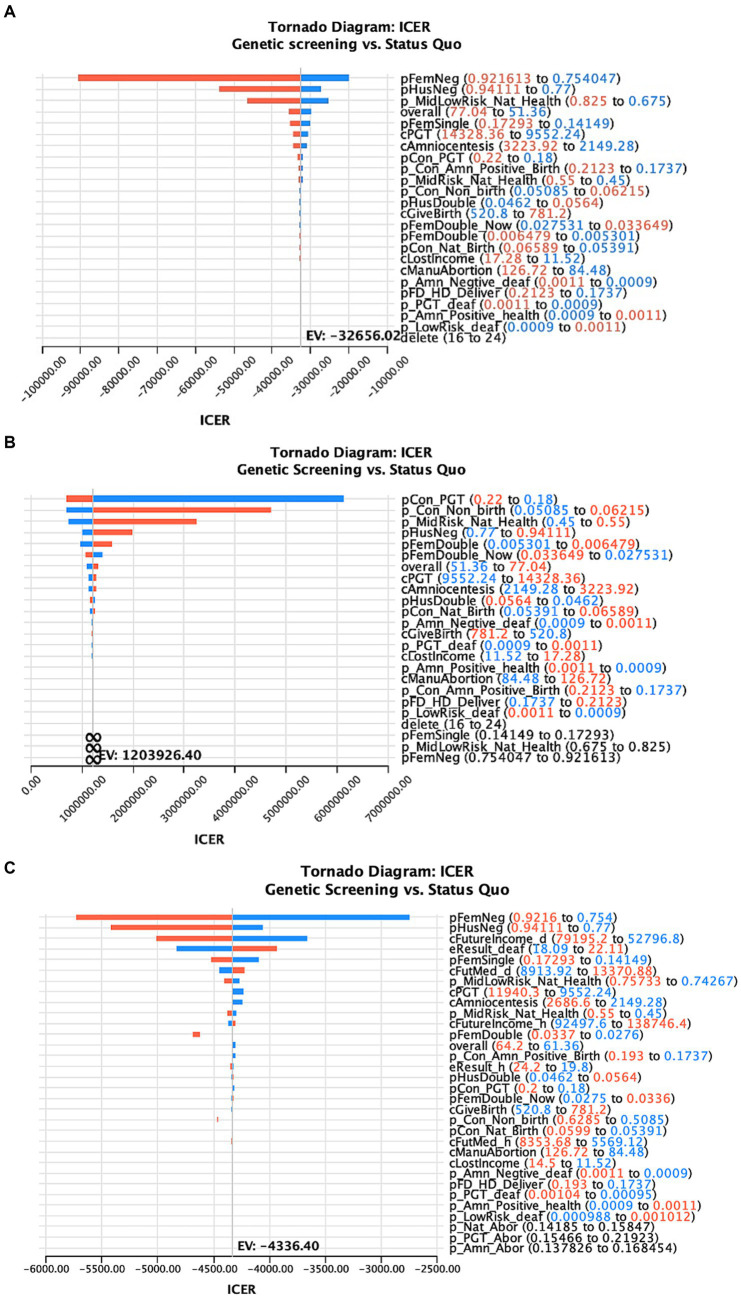
One-way sensitivity analysis of the factors affecting the ICER of deafness screening vs. the status quo of three different models (**A–C** represents the results of the model 1–3 in the main text).

Figure 3 shows the one-way sensitivity analysis of key variables and the ICER distribution in the three models. The variables and results are shown in Supplementary Tables S6–S9.

**Table:** 

Figure 3A	Figure 3B
Figure 3C	

In this study, ART with PGT after a positive deafness genetic screening was the only intervention to simultaneously increase healthy newborns and reduce deaf newborns, compared to the status quo. Therefore, we used a one-way sensitivity analysis to test the effects of this intervention. We found that when the percentage of medium-risk families choosing ART with PGT was >17.4%, there were more healthy than deaf infants compared with the status quo (Figure 4).

**Table:** 

Category	Incremental ICER	Acceptability curve
Model 1	Figure 4A	Figure 4D
Model 2	Figure 4B	Figure 4E
Model 3	Figure 4C	Figure 4F

**Figure:**
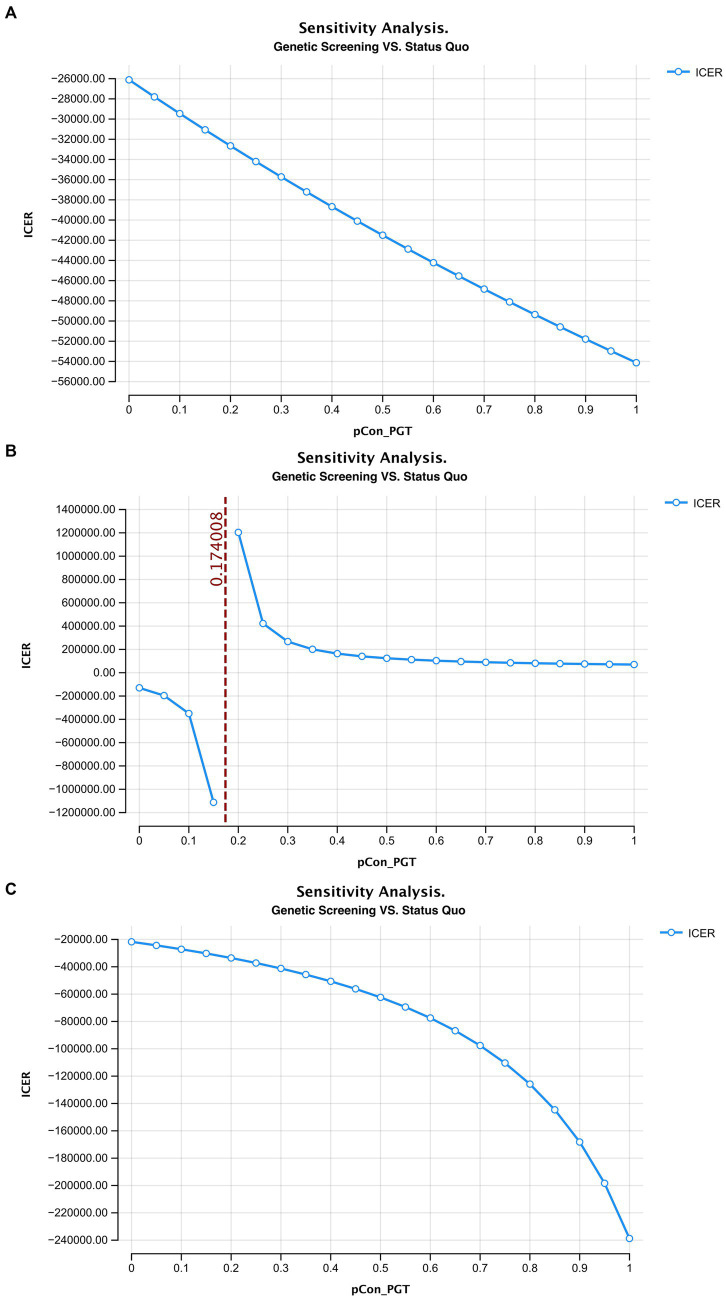

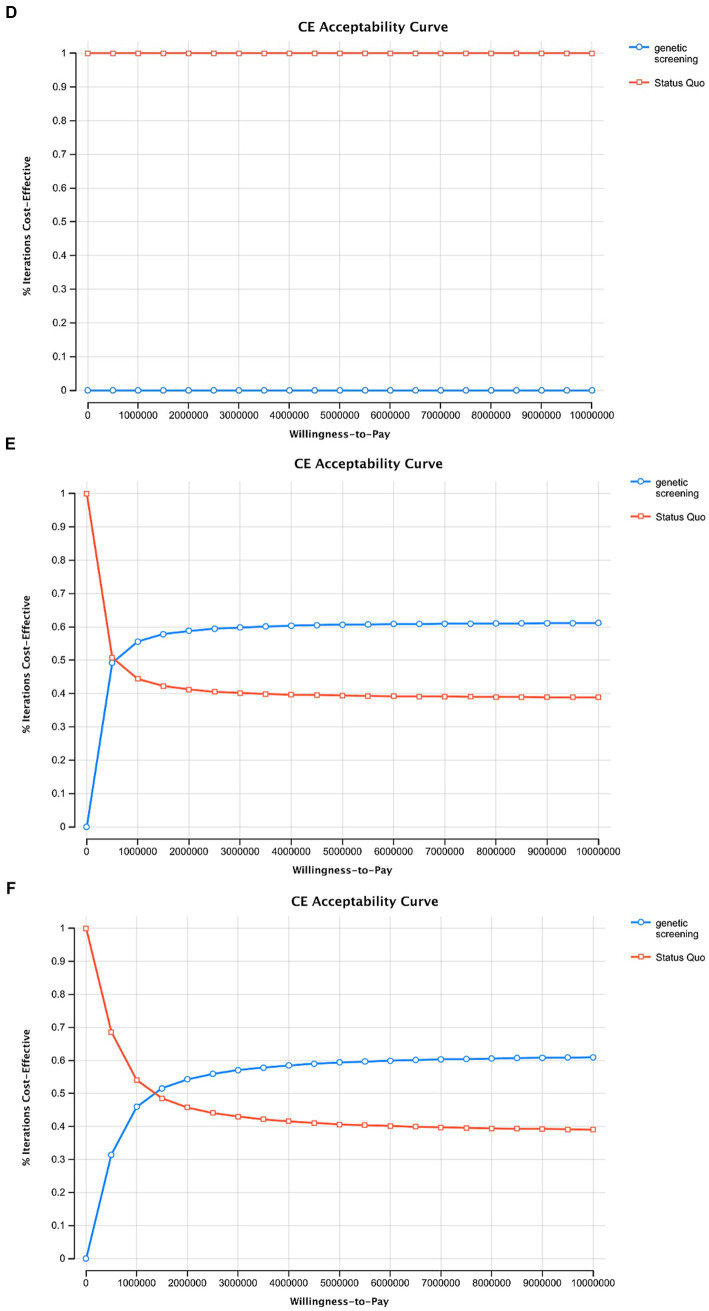
The ICER curve and acceptability curve of deafness screening vs. the status quo of the 3 models (**A–C** represents the ICER of the model 1–3 in the main text, **D–F** represents the acceptability curve of the model 1–3 in the main text).

### Monte Carlo analysis

**Figure 5:**
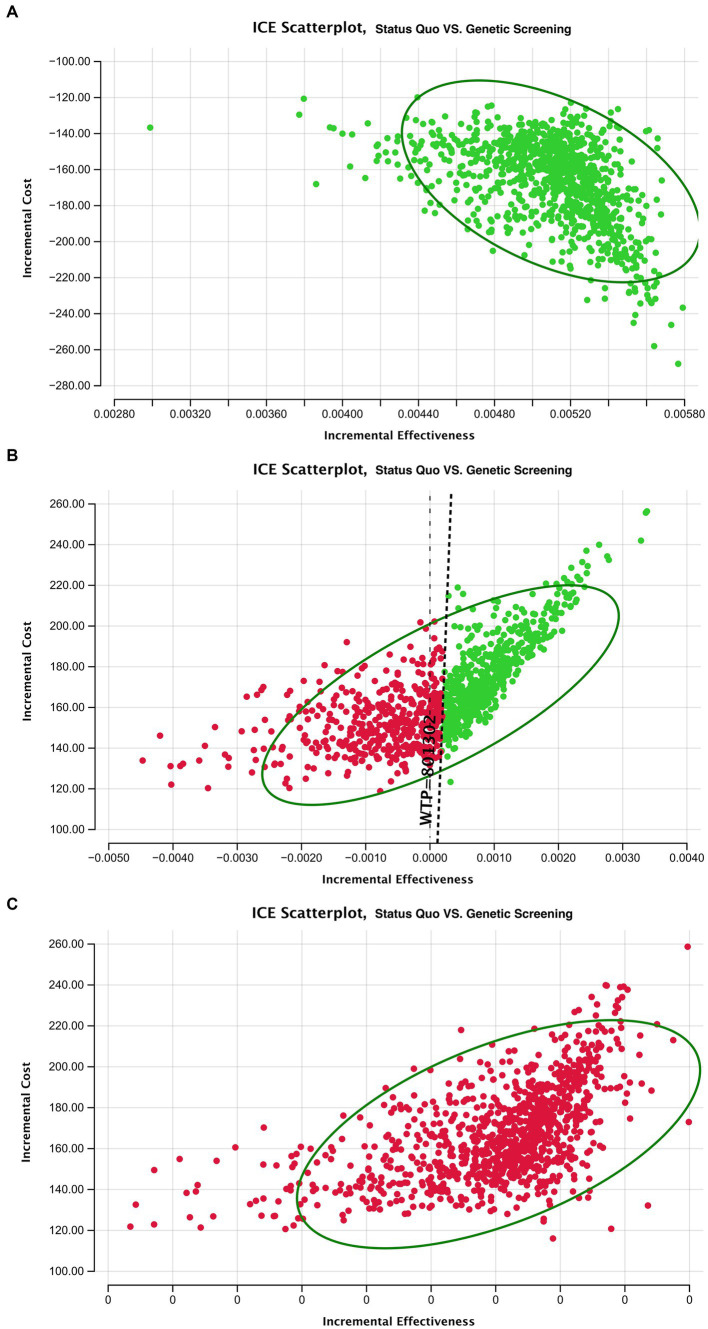
ICER scatterplot of sensitivity analysis for deafness screening strategy vs. the status quo of the three models. Circle means 95% of the ICER results after Monte Carol simulation are located in the circle.

The incremental cost-effectiveness plot is presented in Figure 5. Here, each dot represents an incremental cost plotted against the incremental effectiveness associated with 10,000 Monte Carlo simulations within our 4 models. The simulation falls under a $801,302/life willingness-to-pay threshold. In model 2, nearly half the simulation result fall under the threshold. In models 3 and 4, the deafness screening strategy is not cost-effective.

**Table:** 

Category	ICE Scatterplot
Model 1	Figure 5A
Model 2	Figure 5B
Model 3	Figure 5C

## Discussion

To our knowledge, this study involves the largest sample analysis of the cost-effectiveness of pre-pregnancy genetic screening for deafness in the Chinese population. Our findings help inform decision-making about the implementation of a deafness genetic screening policy. The study cohort was recruited from Shanghai, Beijing, and Suzhou, three of the biggest cities in China, and the sample was representative of the urban population.

We found that deafness screening reduced the birth of deaf newborns with an ICER of $32,656 /case. According to the literature, the ICERs of traditional diagnosis and treatment for childhood hearing loss in developing countries, including the cost of cochlear implants and hearing aid installation, are $15,169 /QALY and $15,430/QALY, respectively (14). The costs of screening for other birth abnormalities also provide us with a useful reference. The ICER of preimplantation genetic testing to prevent the transmission of breast and ovarian cancer (BRCA) is $14,242/QALY for BRCA1 and $12,893/QALY for BRCA2 (21). Another study showed that *in vitro* fertilization preimplantation genetic testing for Huntington’s disease is associated with 77 more QALYs and a cost savings of $46,394,268. Direct comparisons in outcomes cannot be made between a decrease in ICER associated with deafness and other measures that quantify disability metrics because of their different results (life vs. QALY) and the long impact of deafness on the individual, family, and society. However, if we take $53,807 (the sum of the additional medical expenses and productivity loss of deaf individuals) as the opportunity cost, the ICER decrease associated with reducing deaf newborns shows a better effect compared with the additional medical expenses and productivity loss. Furthermore, significant costs are expected to arise following the birth of a deaf newborn, including additional injuries, medical expenditure, and negative occupational effects due to the disability. A systematic review of 59 studies showed that estimates of the economic cost of productivity loss vary widely, from $1.8 to $194 billion in the United States. Excess medical costs resulting from hearing impairment including audiometric testing and treatment with bilateral hearing aids range from $3.3 to $12.8 billion nationally *per annum* in the United States alone (22).

Through a one-way sensitivity analysis, we found that ICER will increase as couples opt for ART with PGT following an adverse genetic finding. In contrast, as couples chose amniocentesis, the ICER decreased. These outcomes are mainly due to the large cost of ART with PGT compared with the relatively low costs of amniocentesis. However, ART can ensure the birth of healthy newborns and reduce the pain of surgical abortion if the amniocentesis test confirms a genetic disorder in the developing fetus (23). Generally speaking, the promotion of pre-pregnancy deafness genetic screening can effectively reduce congenital deafness and the associated familial and societal burdens of disease. The relatively low screening costs ($64.2 in our case) are acceptable to most patients but their widespread coverage by health insurance should be considered as the test can not only reduce the economic burden for patients but also improve the societal effects of screening policies.

We found that when over 17.4% of couples choose ART with PGT, there was a greater proportion of healthy newborns compared to the status quo. A one-way sensitivity analysis showed that with the increase of medium-risk families choosing ART, the ICER of the screening strategy decreased rapidly. The ICER curve tended to be flat around 0.3 and when the ratio was 1 (where all medium-risk families choose ART) the lowest ICER was $69,994.4/case. Therefore, policymakers would need to attempt to increase the proportion of medium-risk families that opt for ART with PGT after screening to reduce the overall societal costs associated with deafness. Several measures could facilitate this goal including the introduction of medical insurance and community education to promote genetic screening and relevant interventions (24, 25).

At present, few studies have focused on the impact of medical screening measures on the wider population. The birth rate is regarded as an important indicator of a country’s development and affects the country’s population size which is one of the most critical factors in policy-making. Therefore, we analyzed the impact of deafness genetic screening policy on the general population in this study. We found that, because screening was more effective in reducing the births of deaf newborns than in promoting the birth of healthy newborns, the overall number of births was reduced. However, reducing the number of deaf newborns will also reduce the cost of social governance (13). In addition, from the families’ perspectives, reducing the births of deaf children can significantly reduce intangible costs that were not considered in this study, such as anxiety, distress, and other psychosocial pressures (26, 27).

From a societal perspective, the deafness genetic screening was not able to increase the QALY compared with the status quo. Relatively speaking, in middle-income countries cochlear implants and hearing aids have been shown to increase QALYs by 5.7 and 4.6, respectively, compared with no treatment (14). Three reasons may explain the differences between these and our findings. First, there is a decrease in newborn births due to genetic screening which leads to a decrease in the overall population QALY. Second, screening benefits a wide range of families with unknown risk of deafness severity of their future child. As a result, utility in this study was derived from a Chinese hearing loss burden of disease database which includes hearing impaired newborns with relatively lower disease severity and better utility in general compared with other age groups. This resulted in the limited QALY improvements for the whole population in general. However, individuals who receive HI and CI always have moderate, severe, or profound deafness. Consequently, HI and CI lead to better effects for their target group compared with the pre-pregnancy screening strategy as a widespread form of population-wide screening (28, 29). Thirdly, in this study we only calculated the total utility of deaf newborns derived from a Chinese hearing loss burden of disease database while the lifetime adverse effects of deafness leading to worse utility were ignored; this may have resulted in excessively conservative results (30–32). If the relationship between the deafness genotype and the severity of the hearing loss is better established, genetic screening becomes more effective because it can be oriented towards identifying and avoiding pathogenic mutations that lead to severe or profound deafness. Focusing on high-risk groups such as consanguineous couples can also improve screening outcomes. A WHO report stated that “Genetic hearing loss is encountered more frequently in children born to consanguineous parents (12–15) and consanguineous marriages are a common tradition in many communities across the world.”

There are some limitations of this study. Firstly, more follow-up research is required to collect follow-up data on couples that opt for various interventions after the screening, which may replace the expert consultant and reference citation in the data collection component and can better help inform policy-makers. Secondly, we utilized estimates of future medical expenditure due to deafness; access to accurate estimates would help make our models more accurate. Thirdly, the severity of the hearing loss is likely to worsen over time. A Markov model as a suitable model for periodic change events could be used to explore the effect of genetic screening and its following interventions with more complicated data and research design. Finally, we did not consider the relationship between cost and the families’ willingness to undergo screening as it is generally believed that better medical insurance coverage and less personal spending will lead to patients’ willingness to receive medical services; this would be valuable for determining medical insurance levels and their effects.

## Data availability statement

The original contributions presented in the study are included in the article/Supplementary material, further inquiries can be directed to the corresponding author.

## Ethics statement

The studies involving humans were approved by the Ethics Committee of Ninth People’s Hospital, Shanghai Jiaotong University School of Medicine. The studies were conducted in accordance with the local legislation and institutional requirements. Written informed consent for participation in this study was provided by the participants’ legal guardians/next of kin.

## Author contributions

YL, ZhiW, ZhaoW, TY and HW made a substantial contribution to the conceptualization and design of the study. YL, YC, and ZhiW collected the data and analysis and interpretation of the data, and drafting of the article. ZhaoW, TY, and YC contributed substantially to the design of the study and drafting and revision of the article. YL, LY, and FC contributed substantially to the interpretation of the data and made critical revisions for the paper. All authors contributed to the article and approved the submitted version.
