# Supplementary material for: A cost-effectiveness analysis of pre-pregnancy genetic screening for deafness: an empirical study in China
Source: Front Public Health. 2023 Dec 7;11:1081339. doi: 10.3389/fpubh.2023.1081339 (PMC10733504; doi:10.3389/fpubh.2023.1081339)
Supplement: Supplementary file 1 [file Table_1.DOCX]

**Supplementary appendix**

[Models Description 2](#_Toc149315746)

[Figure S1 model Schematic 4](#_Toc149315747)

[Figure S2 The Treeage model of deaf related gene screening strategy 4](#_Toc149315748)

[Table S1 45 common mutations for autosomal recessive deafness 5](#_Toc149315749)

[Table S2 Aggregate Age-Specific Mortality Rate per 100,000 Residents and QALY calculated by them 7](#_Toc149315750)

[Table S3 labor productivity and medical expenditure in different age group in terms of healthy and deaf person 8](#_Toc149315751)

[Table S4 the gap of labor productivity and medical expenditure between healthy and deaf person 11](#_Toc149315752)

[Table S5 the result of four Treeage model 12](#_Toc149315753)

[Table S6 Result of one-way sensitivity analysis on probability of having assisted reproductive technologies after screening in model 1(cost effectiveness analysis, deaf=1, health=0) 13](#_Toc149315754)

[Table S7 Result of one-way sensitivity analysis on probability of having assisted reproductive technologies after screening in model 2(cost effectiveness analysis , deaf=0, health=1) 15](#_Toc149315755)

[Table S8 Result of one-way sensitivity analysis on probability of having assisted reproductive technologies after screening in model 3(cost effectiveness analysis deaf=1, health=1) 18](#_Toc149315756)

[Table S9 Result of one-way sensitivity analysis on probability of having assisted reproductive technologies after screening in model 4 (cost utility analysis; deaf=20.1, health=22.1) 22](#_Toc149315757)

[Figure S4 Result of one-way sensitivity analysis on probability of having assisted reproductive technologies after screening in model 1(cost effectiveness analysis, deaf=1, health=0) gene screening VS status quo 25](#_Toc149315758)

[Figure S5 Result of one-way sensitivity analysis on probability of having assisted reproductive technologies after screening in model 1(cost effectiveness analysis, deaf=0, health=1) gene screening VS status quo 26](#_Toc149315759)

[Figure S6 Result of one-way sensitivity analysis on probability of having assisted reproductive technologies after screening in model 1(cost effectiveness analysis, deaf=1, health=1) gene screening VS status quo 27](#_Toc149315760)

[Figure S7 Result of one-way sensitivity analysis on probability of having assisted reproductive technologies after screening in model 1(cost effectiveness analysis, deaf=20.1, health=22.1) gene screening VS status quo 28](#_Toc149315761)

[Table S10 the parameter used for sensitivity analysis and description 29](#_Toc149315762)

[Figure S8 the tornado diagrams of one-way sensitivity analysis in four models 30](#_Toc149315763)

[Figure S9 the cost-effectiveness acceptability curve of the four models 32](#_Toc149315764)

[Table S11 the ICER results of sensitivity analysis in model 1 with all variables 35](#_Toc149315765)

[Table S12 the ICER results of sensitivity analysis in model 2 with all variables 36](#_Toc149315766)

[Table S13 the ICER results of sensitivity analysis in model 3 with all variables 37](#_Toc149315767)

[Table S14 the ICER results of sensitivity analysis in model 4 with all variables 38](#_Toc149315768)

[Table S15 CHEERS 22022 checklist 40](#_Toc149315769)

**Models Description**

In this research, we built four models to describe the effect of deafness genetic screening. We used a two-step screening strategy. In the first step, women planning pregnancy received genetic screening; if negative, their involvement in the study was marked as complete. If pathogenic recessive mutations in the deafness-related genes were identified, their partners were screened in the second step. Based on the results of the genetic screenings of the couples, families were divided into four different risk categories including high-risk, medium-high risk, medium-low risk, and low-risk which reflected their odds of delivering a newborn with genetic hearing loss.

(1) If the screening result shows a high-risk family, only pre-pregnancy medical counseling services were provided regarding the likely hearing loss. The couples were free to decide whether or not to give birth based on this information. (2) if they are Medium-high risk families and Medium-low risk families, the main follow-up interventions included: choosing not to have children; normal pregnancy; normal pregnancy with a prenatal amniocentesis (if amniocentesis screening was positive, couples could decide whether or not to terminate the pregnancy according to the local legal and ethical regulations); utilize assisted reproductive technologies (ART) with a preimplantation genetic test (PGT) and proceed to implantation of fertilized eggs with the desired genotypes. All the choices were designed in the models. (3) if they are (4) Low-risk families, The chance of delivering a child with genetic deafness is relatively low and they can give birth naturally.

Based on the basic model, we built four models to address different research purpose. The difference of these for models is the setting of the result parameter.

In model 1, A cost-effectiveness analysis of taking steps to reduce the birth of deaf newborns. We set the deafness outcome as 1 and the health outcome as 0 and tested the effect of reducing the number of deaf infants. The resulting gap in the model between the screening arm and the status quo arm is the cost-effectiveness of deaf newborns in these two scenarios.

In model 2, A cost-effectiveness analysis of increasing the birth of healthy newborns. We set the health outcome as 1 and the deafness outcome as 0 and tested the impact of the screening strategy on the birth of healthy newborns.

In model 3, A cost-effectiveness analysis was carried to evaluate the effect of overall newborns. We set both the health outcome and the deafness outcome as 1 and tested the impact of the screening strategy on the birth of newborns. In the manuscript, we focus on the effect of screening strategy on deafness newborns and society. So, the results of model 3 was not shown in the main manuscript but in this Supplementary appendix.

In model 4, A cost-utility and policy feasibility analysis of pre-pregnancy deafness genetic screening. The severity of genetic deafness in newborns varies with age. We calculated the health utilization of deaf newborns based on the proportion of different degrees of deafness in China in 2019 from the Chinese global burden of disease data (GBD) multiplied by the utility of different deafness levels from literature, which is 0.91[15]. According to the Chinese life table[16] and the 5% discount rate, the QALY of a healthy person in China is 22.1. Therefore, we set the utility as 22.1 for healthy newborns and 20.1 for deaf newborns.

In the following part, we use model 1 to 4 to represent the different settling of the parameter and their results.

The basic model Schematic and Treeage model were shown in Figure S1 and Figure S2

**Figure S1 model Schematic**


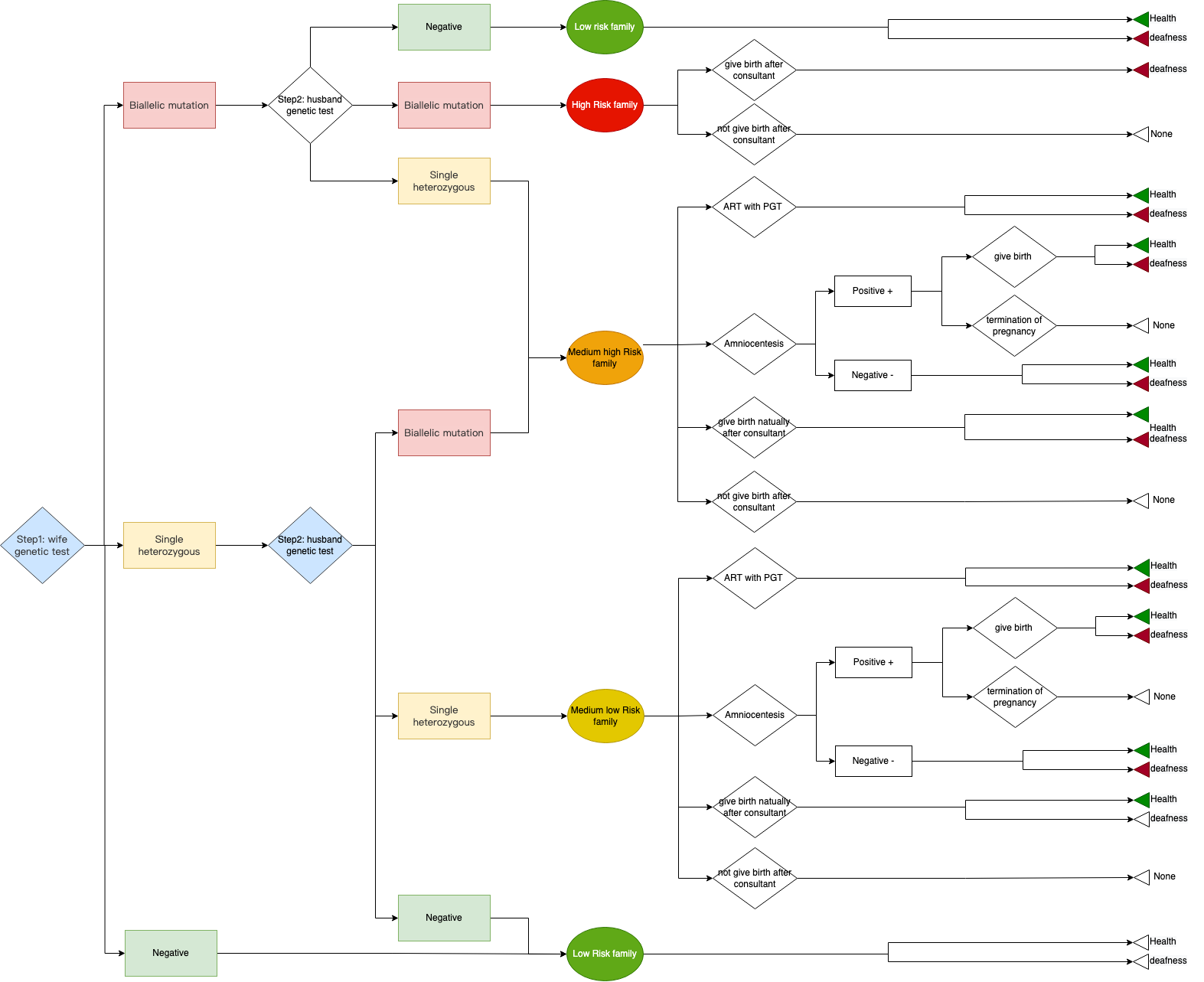


**Figure S2 The Treeage model of deaf related gene screening strategy**


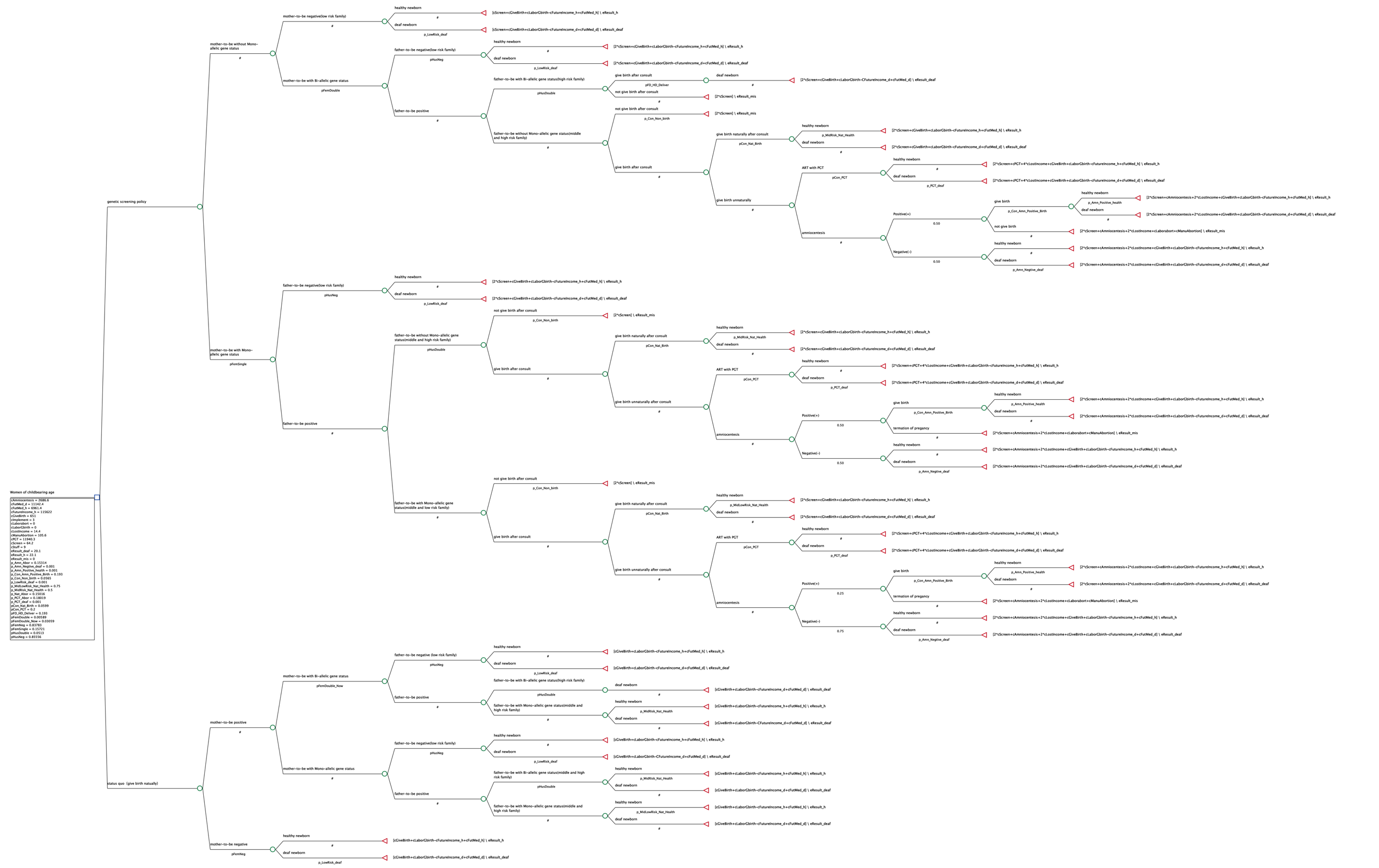


**Table S1 45 common mutations for autosomal recessive deafness**

| **Gene** | **Variation Location** | | **Protein change** |
| --- | --- | --- | --- |
| **GJB2** | 1 | NM_004004.6(GJB2):c.35del | p.Gly12fs |
|  | 2 | NM_004004.6(GJB2):c.176_191del | p.Gly59fs |
|  | 3 | NM_004004.6(GJB2):c.235del | p.Leu79fs |
|  | 4 | NM_004004.6(GJB2):c.299_300del | p.His100fs |
|  | 5 | NM_004004.6(GJB2):c.109G>A | p.Val37Ile |
|  | 6 | NM_004004.6(GJB2):c.35dup | p.Val13fs |
|  | 7 | NM_004004.6(GJB2):c.508_511dup | p.Ala171fs |
|  | 8 | NM_004004.6(GJB2):c.257C>G | p.Thr86Arg |
|  | 9 | NM_004004.6(GJB2):c.571T>C | p.Phe191Leu |
|  | 10 | NM_004004.6(GJB2):c.427C>T | p.Arg143Trp |
|  | 11 | NM_004004.6(GJB2):c.9G>A | p.Trp3Ter |
|  | 12 | NM_004004.6(GJB2):c.344T>G | p.Phe115Cys |
|  | 13 | NM_004004.6(GJB2):c.583A>G | p.Met195Val |
|  | 14 | NM_004004.6(GJB2):c.187G>T | p.Val63Leu |
| **SLC26A4** | 15 | NM_000441.2(SLC26A4):c.919-2A>G | Splice site |
|  | 16 | NM_000441.2(SLC26A4):c.2168A>G | p.His723Arg |
|  | 17 | NM_000441.2(SLC26A4):c.439A>G | p.Met147Val |
|  | 18 | NM_000441.2(SLC26A4):c.1226G>A | p.Arg409His |
|  | 19 | NM_000441.2(SLC26A4):c.916dup | p.Val306fs |
|  | 20 | NM_000441.2(SLC26A4):c.1520del | p.Leu506_Leu507insTer |
|  | 21 | NM_000441.2(SLC26A4):c.2044G>T | p.Glu682Ter |
|  | 22 | NM_000441.2(SLC26A4):c.589G>A | p.Gly197Arg |
|  | 23 | NM_000441.2(SLC26A4):c.1229C>T | p.Thr410Met |
|  | 24 | NM_000441.2(SLC26A4):c.1174A>T | p.Asn392Tyr |
|  | 25 | NM_000441.2(SLC26A4):c.2027T>A | p.Leu676Gln |
|  | 26 | NM_000441.2(SLC26A4):c.1975G>C | p.Val659Leu |
|  | 27 | NM_000441.2(SLC26A4):c.1238A>G | p.Gln413Arg |
|  | 28 | NM_000441.2(SLC26A4):c.1595G>T | p.Ser532Ile |
|  | 29 | NM_000441.2(SLC26A4):c.1079C>T | p.Ala360Val |
|  | 30 | NM_000441.2(SLC26A4):c.1983C>A | p.Asp661Glu |
|  | 31 | NM_000441.2(SLC26A4):c.281C>T | p.Thr94Ile |
|  | 32 | NM_000441.2(SLC26A4):c.1692dup | p.Cys565fs |
|  | 33 | NM_000441.2(SLC26A4):c.1707+5G>A | Splice site |
|  | 34 | NM_000441.2(SLC26A4):c.754T>C | p.Ser252Pro |
|  | 35 | NM_000441.2(SLC26A4):c.1178_1180del | p.Phe394del |
|  | 36 | NM_000441.2(SLC26A4):c.-2071_304+3801del | CNV |
|  | 37 | NM_000441.2(SLC26A4):c.1489G>A | p.Gly497Ser |
|  | 38 | NM_000441.2(SLC26A4):c.1336C>T | p.Gln446Ter |
|  | 39 | NM_000441.2(SLC26A4):c.1548_1549insC | p.Ser517fs |
|  | 40 | NM_000441.2(SLC26A4):c.414del | p.Val138fs |
|  | 41 | NM_000441.2(SLC26A4):c.600+2T>A | Splice site |
|  | 42 | NM_000441.2(SLC26A4):c.1544+9C>T | Splice site |
|  | 43 | NM_000441.2(SLC26A4):c.1829C>A | p.Ser610Ter |
|  | 44 | NM_000441.2(SLC26A4):c.1949T>A | p.Val650Asp |
|  | 45 | NM_000441.2(SLC26A4):c.2343A>G | p.Ter780Trp |

**Table S2 Aggregate Age-Specific Mortality Rate per 100,000 Residents and QALY calculated by them**

| **Age** | **Urban** | **Rural** | **Total** | **Survival rate** | **5%Discount Rate QALY** | **8%Discount RateQALY** |
| --- | --- | --- | --- | --- | --- | --- |
| <1 | 221.81 | 248.16 | 232.19 | 0.9977 | 0.9977 | 0.9977 |
| 1~ | 26.34 | 31.42 | 28.34 | 0.9997 | 3.2899 | 2.9393 |
| 5~ | 15.02 | 19.46 | 16.77 | 0.9998 | 3.9170 | 3.6745 |
| 10~ | 17.54 | 21.79 | 19.21 | 0.9998 | 3.0690 | 2.7008 |
| 15~ | 25.18 | 30.18 | 27.15 | 0.9997 | 2.4044 | 1.9850 |
| 20~ | 26.02 | 37.72 | 30.63 | 0.9997 | 1.8839 | 1.4590 |
| 25~ | 28.16 | 46.8 | 35.50 | 0.9996 | 1.4760 | 1.0724 |
| 30~ | 44.56 | 70.86 | 54.92 | 0.9995 | 1.1563 | 0.7881 |
| 35~ | 68.76 | 96.45 | 79.67 | 0.9992 | 0.9057 | 0.5791 |
| 40~ | 108.4 | 146.37 | 123.36 | 0.9988 | 0.7094 | 0.4255 |
| 45~ | 186.96 | 226.76 | 202.64 | 0.9980 | 0.5554 | 0.3125 |
| 50~ | 270.3 | 376.42 | 312.11 | 0.9969 | 0.4347 | 0.2294 |
| 55~ | 487.86 | 570.55 | 520.44 | 0.9948 | 0.3399 | 0.1683 |
| 60~ | 746.95 | 838.69 | 783.10 | 0.9922 | 0.2656 | 0.1234 |
| 65~ | 1294.15 | 1439.09 | 1351.26 | 0.9865 | 0.2069 | 0.0902 |
| 70~ | 2145.4 | 2660.35 | 2348.29 | 0.9765 | 0.1605 | 0.0656 |
| 75~ | 3840.66 | 4704.96 | 4181.19 | 0.9582 | 0.1234 | 0.0473 |
| 80~ | 6619.27 | 7668.76 | 7032.77 | 0.9297 | 0.0938 | 0.0337 |
| 85~ | 19451.2 | 19718.49 | 19556.51 | 0.8044 | 0.0636 | 0.0215 |
| Total | | | | | 22.0527 | 17.7132 |

The number of deaths for each age group is calculated based on China's urbanization rate of 0.606 in 2021, and is proportionally aggregated into the total mortality rate and survival rate for that age group. Then, the present value QALY for each age group is calculated with the Discount Rate to present (time of birth). Consequently, The total QALY is 22.1.

**Table S3 labor productivity and medical expenditure in different age group in terms of healthy and deaf person**

|  |  | **normal Salary** | | | **disbility Salary** | | | **normal medical expenditure** | | | **disbility medical expenditure** | | |
| --- | --- | --- | --- | --- | --- | --- | --- | --- | --- | --- | --- | --- | --- |
|  | **Age** | **5%Discount Rate** | **0Discount Rate** | **8%Discount Rate** | **5%Discount Rate** | **0Discount Rate** | **8%Discount Rate** | **5%Discount Rate** | **0Discount Rate** | **8%Discount Rate** | **5%Discount Rate** | **0Discount Rate** | **8%Discount Rate** |
| 1 | <1 | 35046.44 | 35046.44 | 35046.44 | 20004.20 | 20004.20 | 20004.20 | 2110.09 | 2110.09 | 2110.09 | 3377.39 | 3377.39 | 3377.39 |
| 2 | 1~ | 115566.81 | 140472.18 | 103251.24 | 65964.54 | 80180.31 | 58934.92 | 6958.09 | 8457.60 | 6216.59 | 11137.07 | 13537.18 | 9950.23 |
| 3 | 5~ | 137595.46 | 175610.55 | 129078.99 | 78538.31 | 100236.99 | 73677.18 | 8284.40 | 10573.23 | 7771.64 | 13259.96 | 16923.44 | 12439.23 |
| 4 | 10~ | 107807.01 | 175606.25 | 94874.59 | 61535.31 | 100234.54 | 54153.60 | 6490.89 | 10572.97 | 5712.25 | 10389.27 | 16923.02 | 9142.98 |
| 5 | 15~ | 84462.91 | 175592.31 | 69730.12 | 48210.70 | 100226.58 | 39801.36 | 5085.37 | 10572.13 | 4198.34 | 8139.62 | 16921.68 | 6719.83 |
| 6 | 20~ | 66176.59 | 175586.20 | 51251.94 | 37773.03 | 100223.10 | 29254.17 | 3984.39 | 10571.76 | 3085.80 | 6377.38 | 16921.09 | 4939.11 |
| 7 | 25~ | 51848.56 | 175577.64 | 37669.87 | 29594.72 | 100218.21 | 21501.64 | 3121.72 | 10571.25 | 2268.04 | 4996.60 | 16920.27 | 3630.21 |
| 8 | 30~ | 40616.82 | 175543.53 | 27683.10 | 23183.73 | 100198.74 | 15801.28 | 2445.47 | 10569.19 | 1666.75 | 3914.21 | 16916.98 | 2667.80 |
| 9 | 35~ | 31816.46 | 175500.07 | 20342.87 | 18160.56 | 100173.93 | 11611.53 | 1915.62 | 10566.57 | 1224.81 | 3066.12 | 16912.79 | 1960.42 |
| 10 | 40~ | 24918.13 | 175423.33 | 14946.08 | 14223.05 | 100130.13 | 8531.09 | 1500.28 | 10561.95 | 899.88 | 2401.34 | 16905.40 | 1440.34 |
| 11 | 45~ | 19508.51 | 175284.08 | 10977.09 | 11135.29 | 100050.65 | 6265.63 | 1174.58 | 10553.57 | 660.91 | 1880.02 | 16891.98 | 1057.85 |
| 12 | 50~ | 15268.66 | 175091.81 | 8059.64 | 8715.22 | 99940.90 | 4600.37 | 919.30 | 10541.99 | 485.26 | 1471.43 | 16873.45 | 776.70 |
| 13 | 55~ | 11938.39 | 174725.90 | 5911.70 | 6814.33 | 99732.04 | 3374.34 | 718.79 | 10519.96 | 355.93 | 1150.49 | 16838.18 | 569.71 |
| 14 | 60~ | 9329.35 | 174264.57 | 4333.80 | 5325.11 | 99468.72 | 2473.70 | 561.70 | 10492.19 | 260.93 | 899.06 | 16793.73 | 417.64 |
| 15 | 65~ | 7267.93 | 173266.65 | 3167.23 | 4148.47 | 98899.12 | 1807.83 | 437.59 | 10432.10 | 190.69 | 700.40 | 16697.56 | 305.22 |
| 16 | 70~ | 5637.06 | 171515.46 | 2304.48 | 3217.58 | 97899.55 | 1315.38 | 339.40 | 10326.67 | 138.75 | 543.24 | 16528.80 | 222.08 |
| 17 | 75~ | 4333.88 | 168296.15 | 1662.07 | 2473.74 | 96062.00 | 948.69 | 260.94 | 10132.84 | 100.07 | 417.65 | 16218.56 | 160.17 |
| 18 | 80~ | 3294.65 | 163287.64 | 1185.31 | 1880.56 | 93203.19 | 676.57 | 198.37 | 9831.28 | 71.37 | 317.50 | 15735.89 | 114.23 |
| 19 | 85~ | 2233.70 | 141290.94 | 753.87 | 1274.97 | 80647.66 | 430.30 | 134.49 | 8506.90 | 45.39 | 215.26 | 13616.09 | 72.65 |
| Total (Chinese Yuan) | | 774667.277 | 774667.28 | 3096981.71 | 622230.43 | 442173.43 | 1767730.57 | 355163.79 | 46641.46 | 186464.25 | 37463.49 | 74654.02 | 298453.47 |
| Total(US Dollar) | | 115621.982 | 115621.98 | 462236.08 | 92870.21 | 65996.03 | 263840.38 | 53009.52 | 6961.41 | 27830.49 | 5591.57 | 11142.39 | 44545.29 |

labor productivity and medical expenditure. According to the "*2019 National Survey Report on Income of Households with Disabilities*", the per capita disposable incomes of Chinese families with disabilities and national residents in 2018 were ¥ 20,050.76 /year and ¥35,128.00/year in 2021 net value, respectively. Based on the per capita disposable income of Chinese residents in 2021 (¥ 35128 /year) from the National Bureau of Statistics of China, the three-year average annual growth rate was calculated to be 7.56% which resulted in the per capita disposable income of Chinese families with disabilities in 2021 to be estimated as $2,992.70.

Because the statistical report is based on the data of whole population in China, the difference of labor productivity and medical expenditure among ages groups is simplified in this research. The labor productivity is obtained by multiplying per capita household income with total QALY

Similarly，According to the "*2019 National Survey Report on Income of Households with Disabilities*", the per capita medical expenditure of Chinese families with disabilities and national residents in 2018 were ¥ 3,385.26/year and ¥2,115/year in 2021net value, respectively. We multiply the medical expenses by the total QALY to obtain the final medical expenses for different populations.

**Table S4 the gap of labor productivity and medical expenditure between healthy and deaf person**

|  |  | **Salary gap** | | | **medical expenditure gap** | | |
| --- | --- | --- | --- | --- | --- | --- | --- |
|  |  | **5%Discount RateSalary** | **0%Discount Ratesalary** | **8%Discount Ratesalary** | **5%Discount Rate** | **0Discount Rate** | **8%Discount Rate** |
| 1 | <1 | 15042.23 | 15042.23 | 15042.23 | -1267.31 | -1267.31 | -1267.31 |
| 2 | 1~ | 49602.27 | 60291.86 | 44316.32 | -4178.98 | -5079.58 | -3733.64 |
| 3 | 5~ | 59057.15 | 75373.55 | 55401.81 | -4975.56 | -6350.21 | -4667.59 |
| 4 | 10~ | 46271.69 | 75371.71 | 40720.99 | -3898.38 | -6350.06 | -3430.74 |
| 5 | 15~ | 36252.20 | 75365.73 | 29928.77 | -3054.24 | -6349.55 | -2521.49 |
| 6 | 20~ | 28403.56 | 75363.11 | 21997.77 | -2393.00 | -6349.33 | -1853.31 |
| 7 | 25~ | 22253.85 | 75359.43 | 16168.23 | -1874.88 | -6349.02 | -1362.17 |
| 8 | 30~ | 17433.09 | 75344.79 | 11881.82 | -1468.73 | -6347.79 | -1001.04 |
| 9 | 35~ | 13655.90 | 75326.14 | 8731.33 | -1150.51 | -6346.22 | -735.61 |
| 10 | 40~ | 10695.07 | 75293.20 | 6414.98 | -901.06 | -6343.44 | -540.46 |
| 11 | 45~ | 8373.22 | 75233.43 | 4711.46 | -705.44 | -6338.41 | -396.94 |
| 12 | 50~ | 6553.44 | 75150.91 | 3459.27 | -552.13 | -6331.45 | -291.44 |
| 13 | 55~ | 5124.06 | 74993.86 | 2537.35 | -431.70 | -6318.22 | -213.77 |
| 14 | 60~ | 4004.24 | 74795.85 | 1860.10 | -337.36 | -6301.54 | -156.71 |
| 15 | 65~ | 3119.46 | 74367.54 | 1359.40 | -262.81 | -6265.45 | -114.53 |
| 16 | 70~ | 2419.47 | 73615.91 | 989.10 | -203.84 | -6202.13 | -83.33 |
| 17 | 75~ | 1860.14 | 72234.15 | 713.37 | -156.72 | -6085.72 | -60.10 |
| 18 | 80~ | 1414.09 | 70084.46 | 508.75 | -119.14 | -5904.61 | -42.86 |
| 19 | 85~ | 958.72 | 60643.29 | 323.57 | -80.77 | -5109.19 | -27.26 |
| Total (Chinese Yuan) | | 332493.847 | 332493.85 | 1329251.14 | 267066.64 | -28012.56 | -111989.21 |

**Table S5 the result of four Treeage model**

|  | Dominance | Strategy | Cost | Incr Cost | Eff | Incr Eff | ICER | NMB |
| --- | --- | --- | --- | --- | --- | --- | --- | --- |
| Deaf=1 health=0 | undominated | Status Quo | 651 |  | 0.00732127 |  |  | 21312.8067 |
|  | abs. dominated | Genetic Screening | 817.603492 | 166.603492 | 0.0022195 | -0.0051018 | -32656.018 | 5840.89301 |
| Deaf=0 health=1 | undominated | Status Quo | 651 |  | 0.99267873 |  |  | 2977385.19 |
|  | undominated | Genetic Screening | 817.603492 | 166.603492 | 0.99281711 | 0.0 | 1203926.4 | 2977633.74 |
| Deaf=1 Health=1 | undominated | Status Quo | 651 |  | 1 |  |  | 2999349 |
|  | abs. dominated | Genetic Screening | 817.603492 | 166.603492 | 0.99503661 | -0.0049634 | -33566.495 | 2984292.24 |
| Deaf=0.9 Health=1 | undominated | Status Quo | -107615.66 |  | 22.0853575 |  |  | 6.64E+07 |
|  | abs. dominated | Genetic Screening | -107184.25 | 431.417117 | 21.9858702 | -0.0994873 | -4336.4038 | 6.61E+07 |

**Table S6 Result of one-way sensitivity analysis on probability of having assisted reproductive technologies after screening** **in model 1(cost effectiveness analysis, deaf=1, health=0)**

| pCon_PGT | Strategy | Strategy Index | Cost | Incr Cost | Eff | Incr Eff | ICER | NMB | C/E | Dominance |
| --- | --- | --- | --- | --- | --- | --- | --- | --- | --- | --- |
| 0 | Status Quo | 1 | 651 | 0 | 0.00732127 | 0 | 0 | 21312.8067 | 88919.0126 |  |
| 0 | Genetic Screening | 0 | 778.598854 | 127.598854 | 0.00243414 | -0.0048871 | -26109.17 | 6523.82403 | 319865.968 | Absolute |
| 0.05 | Status Quo | 1 | 651 | 0 | 0.00732127 | 0 | 0 | 21312.8067 | 88919.0126 |  |
| 0.05 | Genetic Screening | 0 | 788.350013 | 137.350013 | 0.00238048 | -0.0049408 | -27799.209 | 6353.09127 | 331172.651 | Absolute |
| 0.1 | Status Quo | 1 | 651 | 0 | 0.00732127 | 0 | 0 | 21312.8067 | 88919.0126 |  |
| 0.1 | Genetic Screening | 0 | 798.101173 | 147.101173 | 0.00232682 | -0.0049944 | -29452.933 | 6182.35852 | 343000.837 | Absolute |
| 0.15 | Status Quo | 1 | 651 | 0 | 0.00732127 | 0 | 0 | 21312.8067 | 88919.0126 |  |
| 0.15 | Genetic Screening | 0 | 807.852333 | 156.852333 | 0.00227316 | -0.0050481 | -31071.5 | 6011.62576 | 355387.46 | Absolute |
| 0.2 | Status Quo | 1 | 651 | 0 | 0.00732127 | 0 | 0 | 21312.8067 | 88919.0126 |  |
| 0.2 | Genetic Screening | 0 | 817.603492 | 166.603492 | 0.0022195 | -0.0051018 | -32656.018 | 5840.89301 | 368373.022 | Absolute |
| 0.25 | Status Quo | 1 | 651 | 0 | 0.00732127 | 0 | 0 | 21312.8067 | 88919.0126 |  |
| 0.25 | Genetic Screening | 0 | 827.354652 | 176.354652 | 0.00216584 | -0.0051554 | -34207.551 | 5670.16026 | 382002.041 | Absolute |
| 0.3 | Status Quo | 1 | 651 | 0 | 0.00732127 | 0 | 0 | 21312.8067 | 88919.0126 |  |
| 0.3 | Genetic Screening | 0 | 837.105812 | 186.105812 | 0.00211218 | -0.0052091 | -35727.118 | 5499.4275 | 396323.559 | Absolute |
| 0.35 | Status Quo | 1 | 651 | 0 | 0.00732127 | 0 | 0 | 21312.8067 | 88919.0126 |  |
| 0.35 | Genetic Screening | 0 | 846.856972 | 195.856972 | 0.00205852 | -0.0052628 | -37215.697 | 5328.69475 | 411391.732 | Absolute |
| 0.4 | Status Quo | 1 | 651 | 0 | 0.00732127 | 0 | 0 | 21312.8067 | 88919.0126 |  |
| 0.4 | Genetic Screening | 0 | 856.608131 | 205.608131 | 0.00200486 | -0.0053164 | -38674.227 | 5157.96199 | 427266.511 | Absolute |
| 0.45 | Status Quo | 1 | 651 | 0 | 0.00732127 | 0 | 0 | 21312.8067 | 88919.0126 |  |
| 0.45 | Genetic Screening | 0 | 866.359291 | 215.359291 | 0.0019512 | -0.0053701 | -40103.608 | 4987.22924 | 444014.447 | Absolute |
| 0.5 | Status Quo | 1 | 651 | 0 | 0.00732127 | 0 | 0 | 21312.8067 | 88919.0126 |  |
| 0.5 | Genetic Screening | 0 | 876.110451 | 225.110451 | 0.00189754 | -0.0054237 | -41504.705 | 4816.49648 | 461709.614 | Absolute |
| 0.55 | Status Quo | 1 | 651 | 0 | 0.00732127 | 0 | 0 | 21312.8067 | 88919.0126 |  |
| 0.55 | Genetic Screening | 0 | 885.86161 | 234.86161 | 0.00184388 | -0.0054774 | -42878.351 | 4645.76373 | 480434.713 | Absolute |
| 0.6 | Status Quo | 1 | 651 | 0 | 0.00732127 | 0 | 0 | 21312.8067 | 88919.0126 |  |
| 0.6 | Genetic Screening | 0 | 895.61277 | 244.61277 | 0.00179021 | -0.0055311 | -44225.342 | 4475.03098 | 500282.357 | Absolute |
| 0.65 | Status Quo | 1 | 651 | 0 | 0.00732127 | 0 | 0 | 21312.8067 | 88919.0126 |  |
| 0.65 | Genetic Screening | 0 | 905.36393 | 254.36393 | 0.00173655 | -0.0055847 | -45546.449 | 4304.29822 | 521356.608 | Absolute |
| 0.7 | Status Quo | 1 | 651 | 0 | 0.00732127 | 0 | 0 | 21312.8067 | 88919.0126 |  |
| 0.7 | Genetic Screening | 0 | 915.11509 | 264.11509 | 0.00168289 | -0.0056384 | -46842.41 | 4133.56547 | 543774.802 | Absolute |
| 0.75 | Status Quo | 1 | 651 | 0 | 0.00732127 | 0 | 0 | 21312.8067 | 88919.0126 |  |
| 0.75 | Genetic Screening | 0 | 924.866249 | 273.866249 | 0.00162923 | -0.005692 | -48113.936 | 3962.83271 | 567669.73 | Absolute |
| 0.8 | Status Quo | 1 | 651 | 0 | 0.00732127 | 0 | 0 | 21312.8067 | 88919.0126 |  |
| 0.8 | Genetic Screening | 0 | 934.617409 | 283.617409 | 0.00157557 | -0.0057457 | -49361.711 | 3792.09996 | 593192.275 | Absolute |
| 0.85 | Status Quo | 1 | 651 | 0 | 0.00732127 | 0 | 0 | 21312.8067 | 88919.0126 |  |
| 0.85 | Genetic Screening | 0 | 944.368569 | 293.368569 | 0.00152191 | -0.0057994 | -50586.396 | 3621.3672 | 620514.6 | Absolute |
| 0.9 | Status Quo | 1 | 651 | 0 | 0.00732127 | 0 | 0 | 21312.8067 | 88919.0126 |  |
| 0.9 | Genetic Screening | 0 | 954.119729 | 303.119729 | 0.00146825 | -0.005853 | -51788.625 | 3450.63445 | 649834.036 | Absolute |
| 0.95 | Status Quo | 1 | 651 | 0 | 0.00732127 | 0 | 0 | 21312.8067 | 88919.0126 |  |
| 0.95 | Genetic Screening | 0 | 963.870888 | 312.870888 | 0.00141459 | -0.0059067 | -52969.01 | 3279.90169 | 681377.856 | Absolute |
| 1 | Status Quo | 1 | 651 | 0 | 0.00732127 | 0 | 0 | 21312.8067 | 88919.0126 |  |
| 1 | Genetic Screening | 0 | 973.622048 | 322.622048 | 0.00136093 | -0.0059603 | -54128.141 | 3109.16894 | 715409.178 | Absolute |

**Table S7 Result of one-way sensitivity analysis on probability of having assisted reproductive technologies after screening in model 2(cost effectiveness analysis , deaf=0, health=1)**

| pCon_PGT | Strategy | Strategy Index | Cost | Incr Cost | Eff | Incr Eff | ICER | NMB | C/E | Dominance |
| --- | --- | --- | --- | --- | --- | --- | --- | --- | --- | --- |
| 0 | Status Quo | 1 | 651 | 0 | 0.99267873 | 0 | 0 | 2977385.19 | 655.801298 |  |
| 0 | Genetic Screening | 0 | 778.598854 | 127.598854 | 0.9916994 | -9.79E-04 | -130291.44 | 2974319.59 | 785.115788 | Absolute |
| 0.05 | Status Quo | 1 | 651 | 0 | 0.99267873 | 0 | 0 | 2977385.19 | 655.801298 |  |
| 0.05 | Genetic Screening | 0 | 788.350013 | 137.350013 | 0.99197883 | -7.00E-04 | -196241.01 | 2975148.13 | 794.724638 | Absolute |
| 0.1 | Status Quo | 1 | 651 | 0 | 0.99267873 | 0 | 0 | 2977385.19 | 655.801298 |  |
| 0.1 | Genetic Screening | 0 | 798.101173 | 147.101173 | 0.99225826 | -4.20E-04 | -349844.94 | 2975976.67 | 804.328075 | Absolute |
| 0.15 | Status Quo | 1 | 651 | 0 | 0.99267873 | 0 | 0 | 2977385.19 | 655.801298 |  |
| 0.15 | Genetic Screening | 0 | 807.852333 | 156.852333 | 0.99253769 | -1.41E-04 | -1112065.5 | 2976805.2 | 813.926106 | Absolute |
| 0.2 | Status Quo | 1 | 651 | 0 | 0.99267873 | 0 | 0 | 2977385.19 | 655.801298 |  |
| 0.2 | Genetic Screening | 0 | 817.603492 | 166.603492 | 0.99281711 | 1.38E-04 | 1203926.4 | 2977633.74 | 823.518733 |  |
| 0.25 | Status Quo | 1 | 651 | 0 | 0.99267873 | 0 | 0 | 2977385.19 | 655.801298 |  |
| 0.25 | Genetic Screening | 0 | 827.354652 | 176.354652 | 0.99309654 | 4.18E-04 | 422090.062 | 2978462.28 | 833.105963 |  |
| 0.3 | Status Quo | 1 | 651 | 0 | 0.99267873 | 0 | 0 | 2977385.19 | 655.801298 |  |
| 0.3 | Genetic Screening | 0 | 837.105812 | 186.105812 | 0.99337597 | 6.97E-04 | 266917.004 | 2979290.81 | 842.687798 |  |
| 0.35 | Status Quo | 1 | 651 | 0 | 0.99267873 | 0 | 0 | 2977385.19 | 655.801298 |  |
| 0.35 | Genetic Screening | 0 | 846.856972 | 195.856972 | 0.9936554 | 9.77E-04 | 200535.126 | 2980119.35 | 852.264245 |  |
| 0.4 | Status Quo | 1 | 651 | 0 | 0.99267873 | 0 | 0 | 2977385.19 | 655.801298 |  |
| 0.4 | Genetic Screening | 0 | 856.608131 | 205.608131 | 0.99393483 | 0.0012561 | 163687.573 | 2980947.89 | 861.835307 |  |
| 0.45 | Status Quo | 1 | 651 | 0 | 0.99267873 | 0 | 0 | 2977385.19 | 655.801298 |  |
| 0.45 | Genetic Screening | 0 | 866.359291 | 215.359291 | 0.99421426 | 0.00153553 | 140250.747 | 2981776.43 | 871.400989 |  |
| 0.5 | Status Quo | 1 | 651 | 0 | 0.99267873 | 0 | 0 | 2977385.19 | 655.801298 |  |
| 0.5 | Genetic Screening | 0 | 876.110451 | 225.110451 | 0.99449369 | 0.00181496 | 124030.541 | 2982604.96 | 880.961296 |  |
| 0.55 | Status Quo | 1 | 651 | 0 | 0.99267873 | 0 | 0 | 2977385.19 | 655.801298 |  |
| 0.55 | Genetic Screening | 0 | 885.86161 | 234.86161 | 0.99477312 | 0.00209439 | 112138.473 | 2983433.5 | 890.516232 |  |
| 0.6 | Status Quo | 1 | 651 | 0 | 0.99267873 | 0 | 0 | 2977385.19 | 655.801298 |  |
| 0.6 | Genetic Screening | 0 | 895.61277 | 244.61277 | 0.99505255 | 0.00237382 | 103046.107 | 2984262.04 | 900.065801 |  |
| 0.65 | Status Quo | 1 | 651 | 0 | 0.99267873 | 0 | 0 | 2977385.19 | 655.801298 |  |
| 0.65 | Genetic Screening | 0 | 905.36393 | 254.36393 | 0.99533198 | 0.00265325 | 95868.8847 | 2985090.57 | 909.610008 |  |
| 0.7 | Status Quo | 1 | 651 | 0 | 0.99267873 | 0 | 0 | 2977385.19 | 655.801298 |  |
| 0.7 | Genetic Screening | 0 | 915.11509 | 264.11509 | 0.99561141 | 0.00293268 | 90059.3726 | 2985919.11 | 919.148858 |  |
| 0.75 | Status Quo | 1 | 651 | 0 | 0.99267873 | 0 | 0 | 2977385.19 | 655.801298 |  |
| 0.75 | Genetic Screening | 0 | 924.866249 | 273.866249 | 0.99589084 | 0.00321211 | 85260.6292 | 2986747.65 | 928.682356 |  |
| 0.8 | Status Quo | 1 | 651 | 0 | 0.99267873 | 0 | 0 | 2977385.19 | 655.801298 |  |
| 0.8 | Genetic Screening | 0 | 934.617409 | 283.617409 | 0.99617027 | 0.00349154 | 81229.9774 | 2987576.18 | 938.210505 |  |
| 0.85 | Status Quo | 1 | 651 | 0 | 0.99267873 | 0 | 0 | 2977385.19 | 655.801298 |  |
| 0.85 | Genetic Screening | 0 | 944.368569 | 293.368569 | 0.9964497 | 0.00377097 | 77796.6701 | 2988404.72 | 947.733309 |  |
| 0.9 | Status Quo | 1 | 651 | 0 | 0.99267873 | 0 | 0 | 2977385.19 | 655.801298 |  |
| 0.9 | Genetic Screening | 0 | 954.119729 | 303.119729 | 0.99672913 | 0.0040504 | 74837.078 | 2989233.26 | 957.250775 |  |
| 0.95 | Status Quo | 1 | 651 | 0 | 0.99267873 | 0 | 0 | 2977385.19 | 655.801298 |  |
| 0.95 | Genetic Screening | 0 | 963.870888 | 312.870888 | 0.99700856 | 0.00432982 | 72259.4862 | 2990061.8 | 966.762906 |  |
| 1 | Status Quo | 1 | 651 | 0 | 0.99267873 | 0 | 0 | 2977385.19 | 655.801298 |  |
| 1 | Genetic Screening | 0 | 973.622048 | 322.622048 | 0.99728798 | 0.00460925 | 69994.42 | 2990890.33 | 976.269706 |  |

**Table S8 Result of one-way sensitivity analysis on probability of having assisted reproductive technologies after screening in model 3(cost effectiveness analysis deaf=1, health=1)**

| pCon_PGT | Strategy | Strategy Index | Cost | Incr Cost | Eff | Incr Eff | ICER | NMB | C/E | Dominance |
| --- | --- | --- | --- | --- | --- | --- | --- | --- | --- | --- |
| 0 | Status Quo | 1 | 651 | 0 | 1 | 0 | 0 | 2999349 | 651 |  |
| 0 | Genetic Screening | 0 | 778.598854 | 127.598854 | 0.99413354 | -0.0058665 | -21750.563 | 2981622.01 | 783.193428 | Absolute |
| 0.05 | Status Quo | 1 | 651 | 0 | 1 | 0 | 0 | 2999349 | 651 |  |
| 0.05 | Genetic Screening | 0 | 788.350013 | 137.350013 | 0.99435931 | -0.0056407 | -24349.846 | 2982289.57 | 792.822079 | Absolute |
| 0.1 | Status Quo | 1 | 651 | 0 | 1 | 0 | 0 | 2999349 | 651 |  |
| 0.1 | Genetic Screening | 0 | 798.101173 | 147.101173 | 0.99458508 | -0.0054149 | -27165.878 | 2982957.13 | 802.446359 | Absolute |
| 0.15 | Status Quo | 1 | 651 | 0 | 1 | 0 | 0 | 2999349 | 651 |  |
| 0.15 | Genetic Screening | 0 | 807.852333 | 156.852333 | 0.99481084 | -0.0051892 | -30226.948 | 2983624.68 | 812.066271 | Absolute |
| 0.2 | Status Quo | 1 | 651 | 0 | 1 | 0 | 0 | 2999349 | 651 |  |
| 0.2 | Genetic Screening | 0 | 817.603492 | 166.603492 | 0.99503661 | -0.0049634 | -33566.495 | 2984292.24 | 821.681817 | Absolute |
| 0.25 | Status Quo | 1 | 651 | 0 | 1 | 0 | 0 | 2999349 | 651 |  |
| 0.25 | Genetic Screening | 0 | 827.354652 | 176.354652 | 0.99526238 | -0.0047376 | -37224.331 | 2984959.79 | 831.293001 | Absolute |
| 0.3 | Status Quo | 1 | 651 | 0 | 1 | 0 | 0 | 2999349 | 651 |  |
| 0.3 | Genetic Screening | 0 | 837.105812 | 186.105812 | 0.99548815 | -0.0045118 | -41248.237 | 2985627.35 | 840.899825 | Absolute |
| 0.35 | Status Quo | 1 | 651 | 0 | 1 | 0 | 0 | 2999349 | 651 |  |
| 0.35 | Genetic Screening | 0 | 846.856972 | 195.856972 | 0.99571392 | -0.0042861 | -45696.061 | 2986294.9 | 850.502292 | Absolute |
| 0.4 | Status Quo | 1 | 651 | 0 | 1 | 0 | 0 | 2999349 | 651 |  |
| 0.4 | Genetic Screening | 0 | 856.608131 | 205.608131 | 0.99593969 | -0.0040603 | -50638.516 | 2986962.46 | 860.100407 | Absolute |
| 0.45 | Status Quo | 1 | 651 | 0 | 1 | 0 | 0 | 2999349 | 651 |  |
| 0.45 | Genetic Screening | 0 | 866.359291 | 215.359291 | 0.99616546 | -0.0038345 | -56162.972 | 2987630.01 | 869.69417 | Absolute |
| 0.5 | Status Quo | 1 | 651 | 0 | 1 | 0 | 0 | 2999349 | 651 |  |
| 0.5 | Genetic Screening | 0 | 876.110451 | 225.110451 | 0.99639123 | -0.0036088 | -62378.661 | 2988297.57 | 879.283586 | Absolute |
| 0.55 | Status Quo | 1 | 651 | 0 | 1 | 0 | 0 | 2999349 | 651 |  |
| 0.55 | Genetic Screening | 0 | 885.86161 | 234.86161 | 0.996617 | -0.003383 | -69423.972 | 2988965.12 | 888.868657 | Absolute |
| 0.6 | Status Quo | 1 | 651 | 0 | 1 | 0 | 0 | 2999349 | 651 |  |
| 0.6 | Genetic Screening | 0 | 895.61277 | 244.61277 | 0.99684276 | -0.0031572 | -77476.88 | 2989632.68 | 898.449387 | Absolute |
| 0.65 | Status Quo | 1 | 651 | 0 | 1 | 0 | 0 | 2999349 | 651 |  |
| 0.65 | Genetic Screening | 0 | 905.36393 | 254.36393 | 0.99706853 | -0.0029315 | -86770.19 | 2990300.24 | 908.025777 | Absolute |
| 0.7 | Status Quo | 1 | 651 | 0 | 1 | 0 | 0 | 2999349 | 651 |  |
| 0.7 | Genetic Screening | 0 | 915.11509 | 264.11509 | 0.9972943 | -0.0027057 | -97614.403 | 2990967.79 | 917.597832 | Absolute |
| 0.75 | Status Quo | 1 | 651 | 0 | 1 | 0 | 0 | 2999349 | 651 |  |
| 0.75 | Genetic Screening | 0 | 924.866249 | 273.866249 | 0.99752007 | -0.0024799 | -110433.1 | 2991635.35 | 927.165554 | Absolute |
| 0.8 | Status Quo | 1 | 651 | 0 | 1 | 0 | 0 | 2999349 | 651 |  |
| 0.8 | Genetic Screening | 0 | 934.617409 | 283.617409 | 0.99774584 | -0.0022542 | -125819.54 | 2992302.9 | 936.728946 | Absolute |
| 0.85 | Status Quo | 1 | 651 | 0 | 1 | 0 | 0 | 2999349 | 651 |  |
| 0.85 | Genetic Screening | 0 | 944.368569 | 293.368569 | 0.99797161 | -0.0020284 | -144631.15 | 2992970.46 | 946.288011 | Absolute |
| 0.9 | Status Quo | 1 | 651 | 0 | 1 | 0 | 0 | 2999349 | 651 |  |
| 0.9 | Genetic Screening | 0 | 954.119729 | 303.119729 | 0.99819738 | -0.0018026 | -168154.86 | 2993638.01 | 955.842752 | Absolute |
| 0.95 | Status Quo | 1 | 651 | 0 | 1 | 0 | 0 | 2999349 | 651 |  |
| 0.95 | Genetic Screening | 0 | 963.870888 | 312.870888 | 0.99842315 | -0.0015769 | -198414.67 | 2994305.57 | 965.393172 | Absolute |
| 1 | Status Quo | 1 | 651 | 0 | 1 | 0 | 0 | 2999349 | 651 |  |
| 1 | Genetic Screening | 0 | 973.622048 | 322.622048 | 0.99864892 | -0.0013511 | -238787.43 | 2994973.12 | 974.939274 | Absolute |

**Table S9 Result of one-way sensitivity analysis on probability of having assisted reproductive technologies after screening in model 4 (cost utility analysis; deaf=20.1, health=22.1)**

| pCon_PGT | Strategy | Strategy Index | Cost | Incr Cost | Eff | Incr Eff | ICER | NMB | C/E | Dominance |
| --- | --- | --- | --- | --- | --- | --- | --- | --- | --- | --- |
| 0 | Status Quo | 1 | -107615.66 | 0 | 22.0853575 | 0 | 0 | 6.64E+07 | -4872.7155 |  |
| 0 | Genetic Screening | 0 | -107113.57 | 502.090449 | 21.9654829 | -0.1198746 | -4188.4656 | 6.60E+07 | -4876.4498 | Absolute |
| 0.05 | Status Quo | 1 | -107615.66 | 0 | 22.0853575 | 0 | 0 | 6.64E+07 | -4872.7155 |  |
| 0.05 | Genetic Screening | 0 | -107131.24 | 484.422116 | 21.9705797 | -0.1147777 | -4220.5231 | 6.60E+07 | -4876.1227 | Absolute |
| 0.1 | Status Quo | 1 | -107615.66 | 0 | 22.0853575 | 0 | 0 | 6.64E+07 | -4872.7155 |  |
| 0.1 | Genetic Screening | 0 | -107148.91 | 466.753783 | 21.9756765 | -0.1096809 | -4255.5601 | 6.60E+07 | -4875.7958 | Absolute |
| 0.15 | Status Quo | 1 | -107615.66 | 0 | 22.0853575 | 0 | 0 | 6.64E+07 | -4872.7155 |  |
| 0.15 | Genetic Screening | 0 | -107166.58 | 449.08545 | 21.9807733 | -0.1045841 | -4294.012 | 6.60E+07 | -4875.469 | Absolute |
| 0.2 | Status Quo | 1 | -107615.66 | 0 | 22.0853575 | 0 | 0 | 6.64E+07 | -4872.7155 |  |
| 0.2 | Genetic Screening | 0 | -107184.25 | 431.417117 | 21.9858702 | -0.0994873 | -4336.4038 | 6.61E+07 | -4875.1424 | Absolute |
| 0.25 | Status Quo | 1 | -107615.66 | 0 | 22.0853575 | 0 | 0 | 6.64E+07 | -4872.7155 |  |
| 0.25 | Genetic Screening | 0 | -107201.92 | 413.748784 | 21.990967 | -0.0943905 | -4383.3736 | 6.61E+07 | -4874.8159 | Absolute |
| 0.3 | Status Quo | 1 | -107615.66 | 0 | 22.0853575 | 0 | 0 | 6.64E+07 | -4872.7155 |  |
| 0.3 | Genetic Screening | 0 | -107219.58 | 396.080451 | 21.9960638 | -0.0892937 | -4435.7054 | 6.61E+07 | -4874.4896 | Absolute |
| 0.35 | Status Quo | 1 | -107615.66 | 0 | 22.0853575 | 0 | 0 | 6.64E+07 | -4872.7155 |  |
| 0.35 | Genetic Screening | 0 | -107237.25 | 378.412118 | 22.0011606 | -0.0841969 | -4494.373 | 6.61E+07 | -4874.1634 | Absolute |
| 0.4 | Status Quo | 1 | -107615.66 | 0 | 22.0853575 | 0 | 0 | 6.64E+07 | -4872.7155 |  |
| 0.4 | Genetic Screening | 0 | -107254.92 | 360.743785 | 22.0062574 | -0.0791001 | -4560.6011 | 6.61E+07 | -4873.8374 | Absolute |
| 0.45 | Status Quo | 1 | -107615.66 | 0 | 22.0853575 | 0 | 0 | 6.64E+07 | -4872.7155 |  |
| 0.45 | Genetic Screening | 0 | -107272.59 | 343.075452 | 22.0113542 | -0.0740032 | -4635.9519 | 6.61E+07 | -4873.5115 | Absolute |
| 0.5 | Status Quo | 1 | -107615.66 | 0 | 22.0853575 | 0 | 0 | 6.64E+07 | -4872.7155 |  |
| 0.5 | Genetic Screening | 0 | -107290.26 | 325.407119 | 22.016451 | -0.0689064 | -4722.4496 | 6.62E+07 | -4873.1858 | Absolute |
| 0.55 | Status Quo | 1 | -107615.66 | 0 | 22.0853575 | 0 | 0 | 6.64E+07 | -4872.7155 |  |
| 0.55 | Genetic Screening | 0 | -107307.93 | 307.738786 | 22.0215478 | -0.0638096 | -4822.7653 | 6.62E+07 | -4872.8603 | Absolute |
| 0.6 | Status Quo | 1 | -107615.66 | 0 | 22.0853575 | 0 | 0 | 6.64E+07 | -4872.7155 |  |
| 0.6 | Genetic Screening | 0 | -107325.59 | 290.070453 | 22.0266447 | -0.0587128 | -4940.4977 | 6.62E+07 | -4872.5349 | Absolute |
| 0.65 | Status Quo | 1 | -107615.66 | 0 | 22.0853575 | 0 | 0 | 6.64E+07 | -4872.7155 |  |
| 0.65 | Genetic Screening | 0 | -107343.26 | 272.40212 | 22.0317415 | -0.053616 | -5080.6138 | 6.62E+07 | -4872.2096 | Absolute |
| 0.7 | Status Quo | 1 | -107615.66 | 0 | 22.0853575 | 0 | 0 | 6.64E+07 | -4872.7155 |  |
| 0.7 | Genetic Screening | 0 | -107360.93 | 254.733787 | 22.0368383 | -0.0485192 | -5250.1675 | 6.62E+07 | -4871.8845 | Absolute |
| 0.75 | Status Quo | 1 | -107615.66 | 0 | 22.0853575 | 0 | 0 | 6.64E+07 | -4872.7155 |  |
| 0.75 | Genetic Screening | 0 | -107378.6 | 237.065454 | 22.0419351 | -0.0434224 | -5459.5248 | 6.62E+07 | -4871.5595 | Absolute |
| 0.8 | Status Quo | 1 | -107615.66 | 0 | 22.0853575 | 0 | 0 | 6.64E+07 | -4872.7155 |  |
| 0.8 | Genetic Screening | 0 | -107396.27 | 219.397121 | 22.0470319 | -0.0383255 | -5724.5659 | 6.62E+07 | -4871.2347 | Absolute |
| 0.85 | Status Quo | 1 | -107615.66 | 0 | 22.0853575 | 0 | 0 | 6.64E+07 | -4872.7155 |  |
| 0.85 | Genetic Screening | 0 | -107413.94 | 201.728788 | 22.0521287 | -0.0332287 | -6070.9139 | 6.63E+07 | -4870.9101 | Absolute |
| 0.9 | Status Quo | 1 | -107615.66 | 0 | 22.0853575 | 0 | 0 | 6.64E+07 | -4872.7155 |  |
| 0.9 | Genetic Screening | 0 | -107431.6 | 184.060455 | 22.0572255 | -0.0281319 | -6542.7616 | 6.63E+07 | -4870.5856 | Absolute |
| 0.95 | Status Quo | 1 | -107615.66 | 0 | 22.0853575 | 0 | 0 | 6.64E+07 | -4872.7155 |  |
| 0.95 | Genetic Screening | 0 | -107449.27 | 166.392122 | 22.0623224 | -0.0230351 | -7223.4139 | 6.63E+07 | -4870.2612 | Absolute |
| 1 | Status Quo | 1 | -107615.66 | 0 | 22.0853575 | 0 | 0 | 6.64E+07 | -4872.7155 |  |
| 1 | Genetic Screening | 0 | -107466.94 | 148.723789 | 22.0674192 | -0.0179383 | -8290.854 | 6.63E+07 | -4869.937 | Absolute |

**Figure S4 Result of one-way sensitivity analysis on probability of having assisted reproductive technologies after screening in model 1(cost effectiveness analysis, deaf=1, health=0) gene screening VS status quo**

 Figure S3-a

 Figure S3-b

Figure S3-c

Figure a to c represent the result of ICER, increment cost and increment effect with the change of probability of having assisted reproductive technologies after screening, respectively.

**Figure S5 Result of one-way sensitivity analysis on probability of having assisted reproductive technologies after screening in model 1(cost effectiveness analysis, deaf=0, health=1) gene screening VS status quo**

 Figure S3-a

 Figure S3-b

Figure S3-c

Figure a to c represent the result of ICER, increment cost and increment effect with the change of probability of having assisted reproductive technologies after screening, respectively.

**Figure S6 Result of one-way sensitivity analysis on probability of having assisted reproductive technologies after screening in model 1(cost effectiveness analysis, deaf=1, health=1) gene screening VS status quo**

Figure S3-a

 Figure S3-b

Figure S3-c

Figure a to c represent the result of ICER, increment cost and increment effect with the change of probability of having assisted reproductive technologies after screening, respectively.

**Figure S7 Result of one-way sensitivity analysis on probability of having assisted reproductive technologies after screening in model 1(cost effectiveness analysis, deaf=20.1, health=22.1) gene screening VS status quo**

Figure S3-a

 Figure S3-b


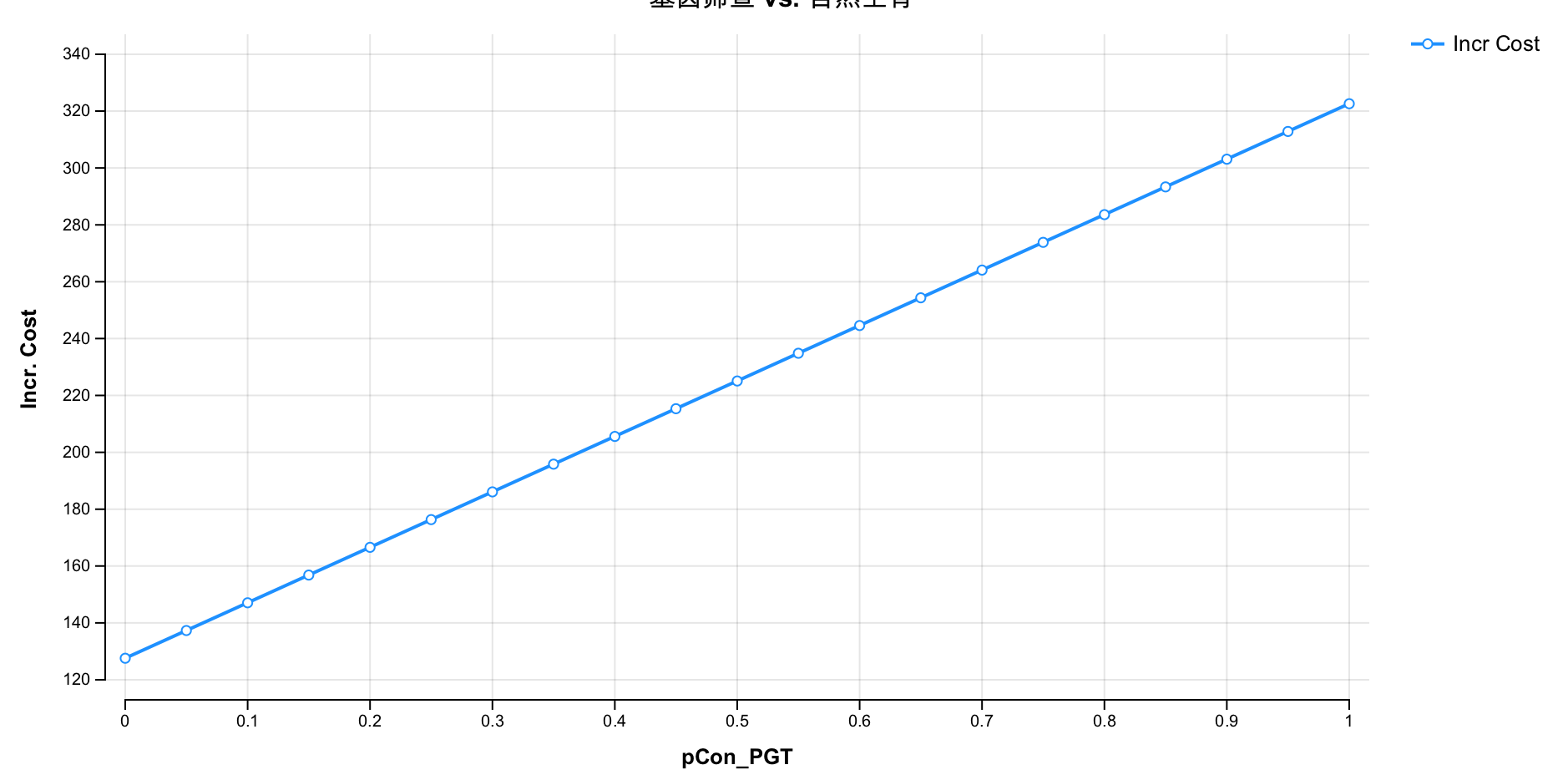
 Figure S3-c

Figure a to c represent the result of ICER, increment cost and increment effect with the change of probability of having assisted reproductive technologies after screening, respectively.

**Table S10 the parameter used for sensitivity analysis and description**

| **Variable Name** | **Variable Description** |
| --- | --- |
| pFemNeg | percentage of wife passes screening |
| pHusNeg | percentage of Husband passes screening |
| p_MidLowRisk_Nat_Health | percentage of Negative amniocentesis in medium-low-risk family |
| cScreen | overall screening costs |
| pFemSingle | percentage of Wife with single heterozygous mutation |
| cPGT | cost of ART with PGT |
| cAmniocentesis | cost of Amniocentesis |
| pCon_PGT | percentage of Medium-risk families choose to take the ART with PGT |
| p_Con_Amn_Positive_Birth | percentage of High-risk families choose to give birth naturally |
| p_MidRisk_Nat_Health | percentage of Medium-high-risk families have healthy newborns naturally |
| p_Con_Non_birth | percentage of Medium-risk families choose not to have children |
| pHusDouble | percengate of husband with Biallelic Mutation |
| cGiveBirth | cost of give birth |
| pFemDouble_Now | percentage of wife with biallelic mutation in the condition of wife positive after screening |
| pFemDouble | percentage of wife with biallelic mutation |
| pCon_Nat_Birth | percentage of High-risk families choose to give birth naturally |
| cLostIncome | Productivity loss of Amniocentesis |
| cManuAbortion | cost of manual abortion |
| p_Amn_Negtive_deaf | percentage of Medium-risk families have healthy newborns with negative amniocentesis |
| pFD_HD_Deliver | percentage of High-risk families choose to give birth naturally |
| p_PGT_deaf | percentage of Medium-risk families have deaf newborns after ART with PGT |
| p_Amn_Positive_health | percentage of Medium-risk families have healthy newborns with positive amniocentesis |
| p_LowRisk_deaf | percentage of High-risk families choose to give birth naturally |

**Figure S8 the tornado diagrams of one-way sensitivity analysis in four models**

Both one-way deterministic and simulated probabilistic sensitivity analyses were conducted to assess the robustness of the main outcomes. A change of plus or minus 10% (for prevalence, utility, sensitivity, specificity, compliance, and transition probability from published literature) and plus or minus 20% (for treatment-related costs provided by real-world screening programmes and hospitals) of the original values for the parameters were used for probability-related data in sensitivity analysis.

Figure S8-a Model 1


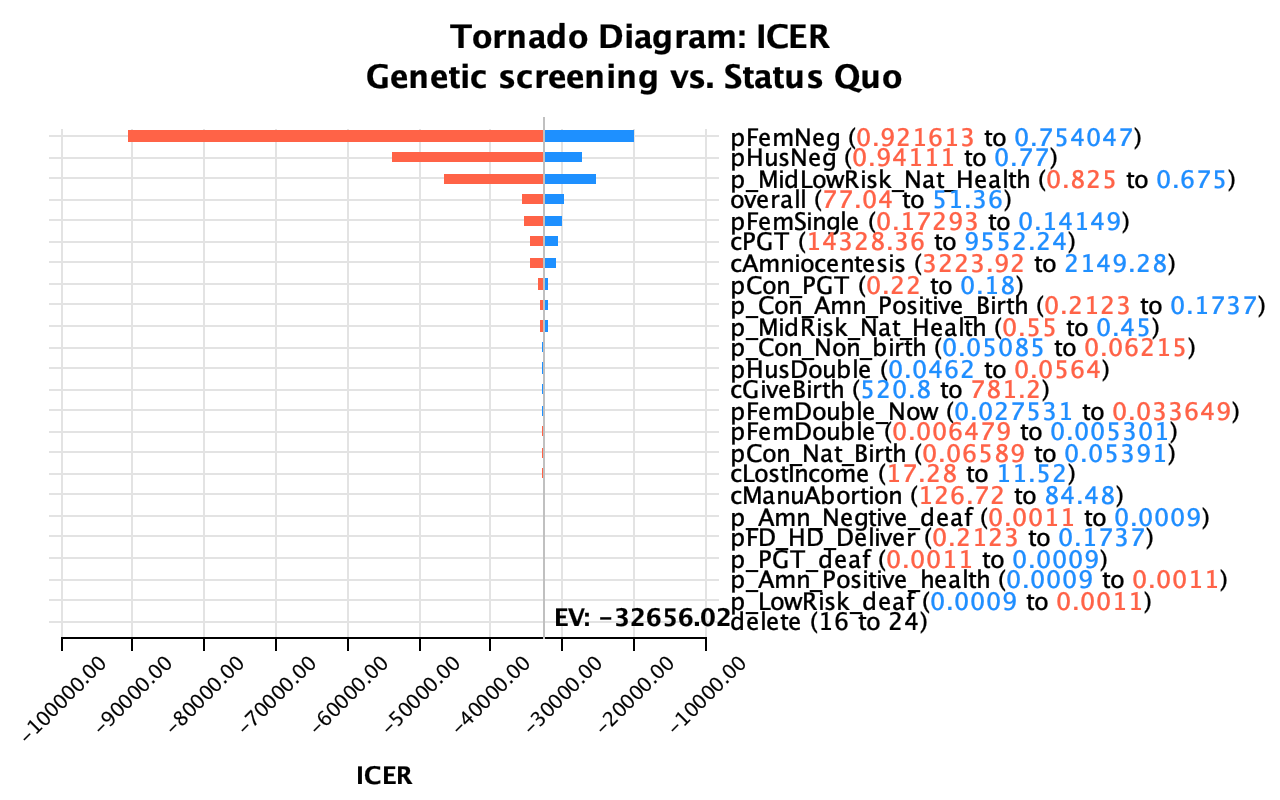


Figure S8-b


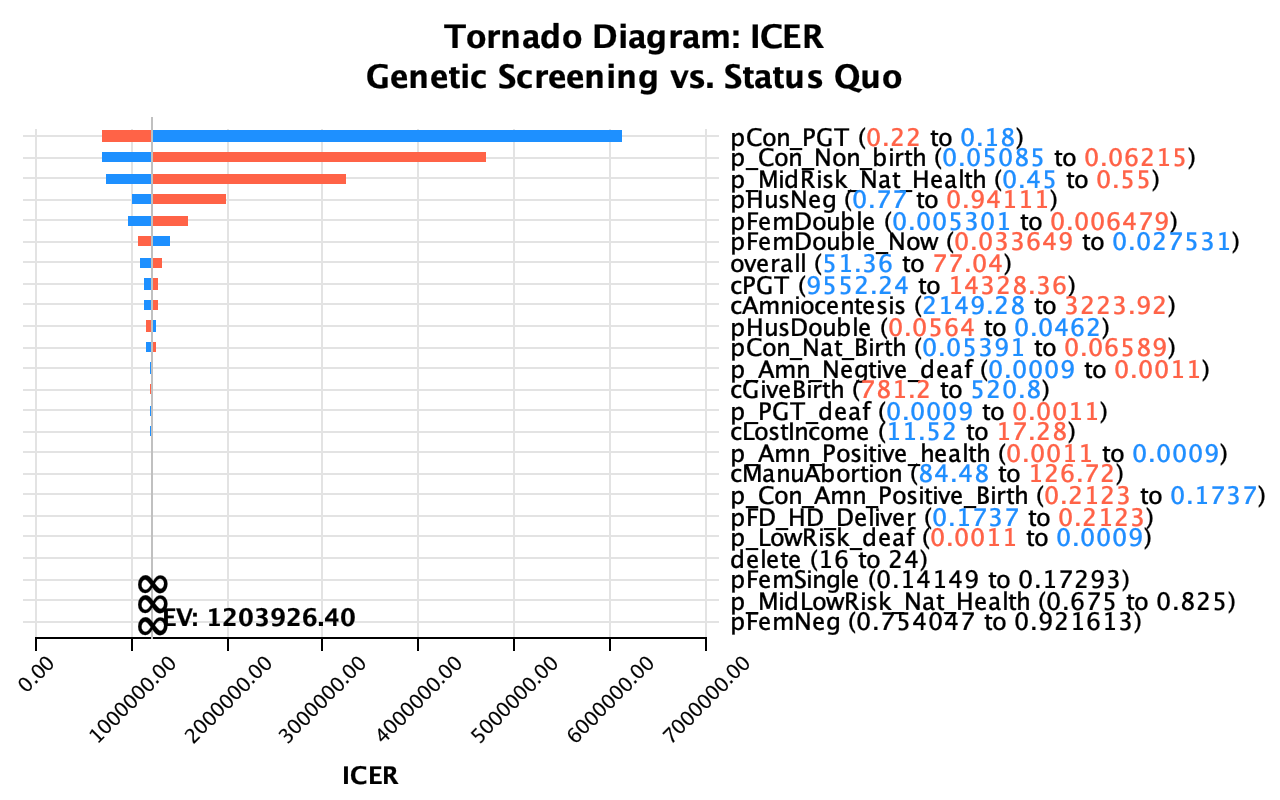


Figure S8-c


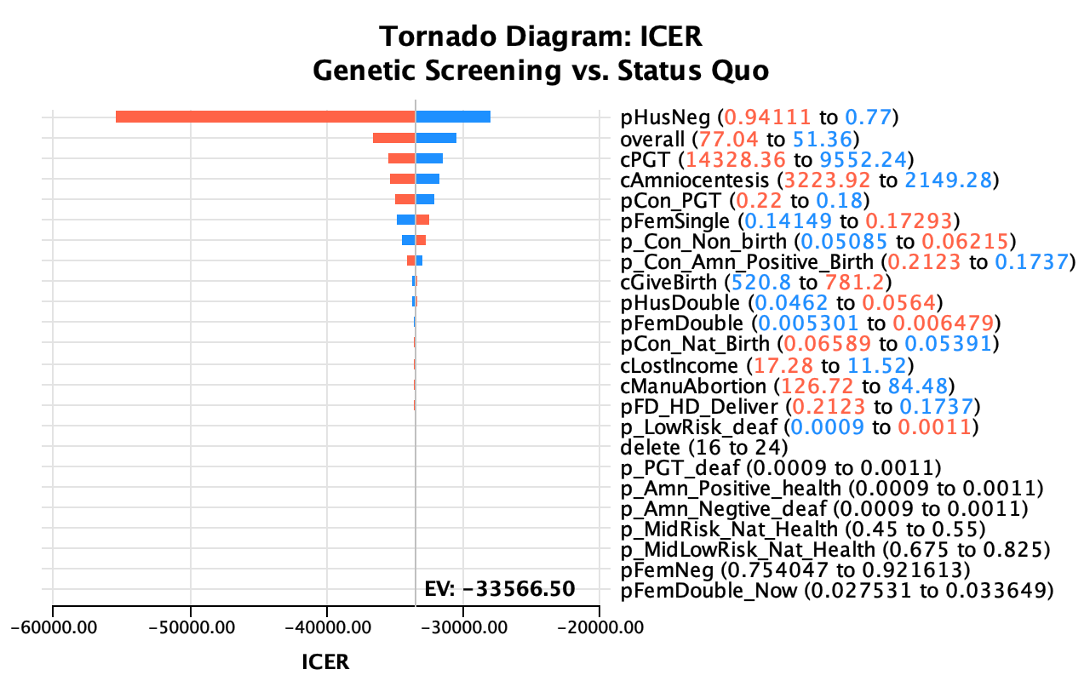


Figure S8-d


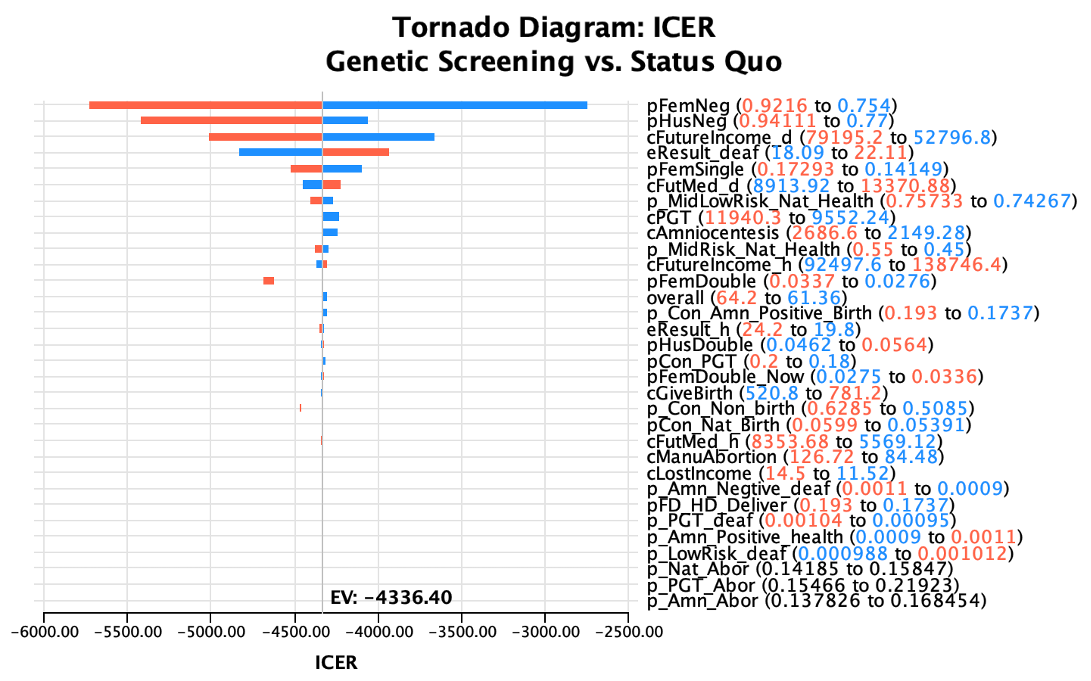


**Figure S9 the cost-effectiveness acceptability curve of the four models**

Figure S9-a Model 1 (Deaf=1 Health=0)


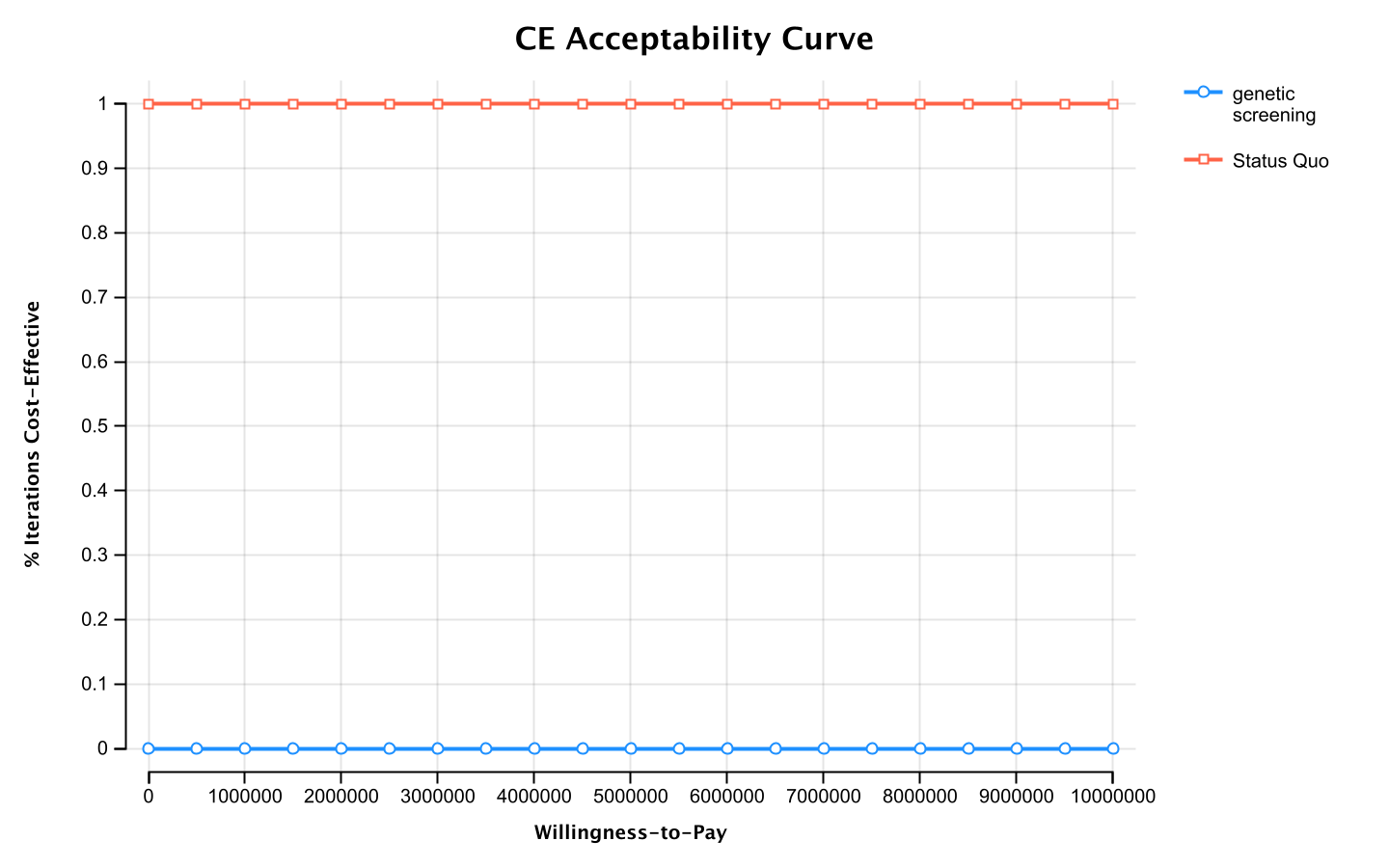


Figure S9-b Model 2 (Deaf=0 Health=1)


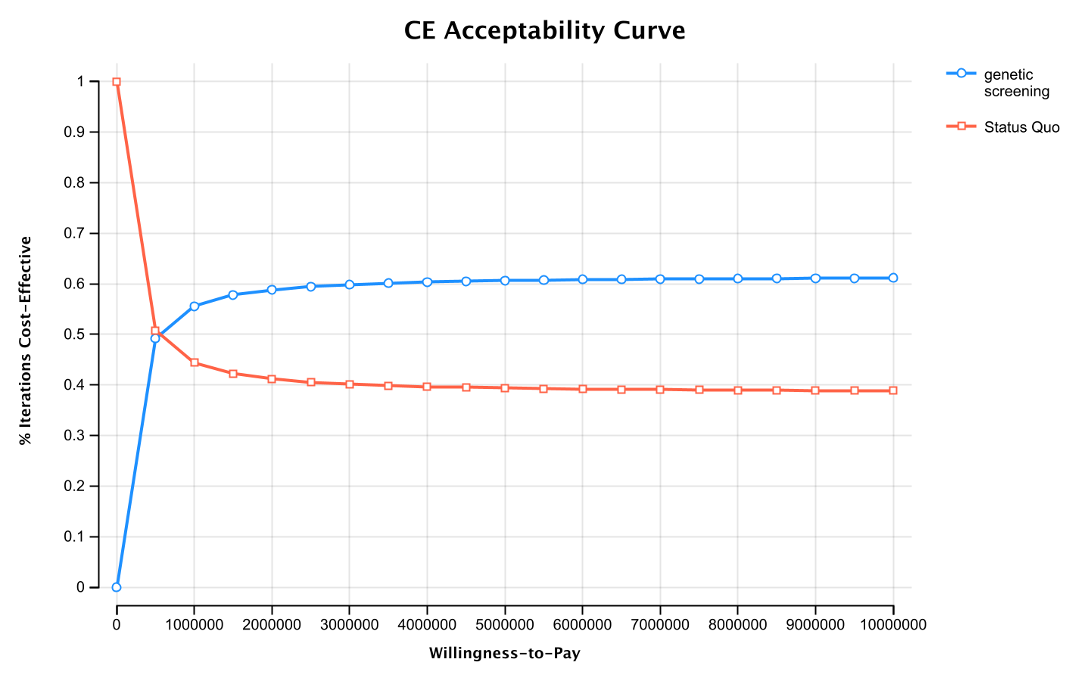


Figure S9-c Model 3 (Deaf=1 Health=1)


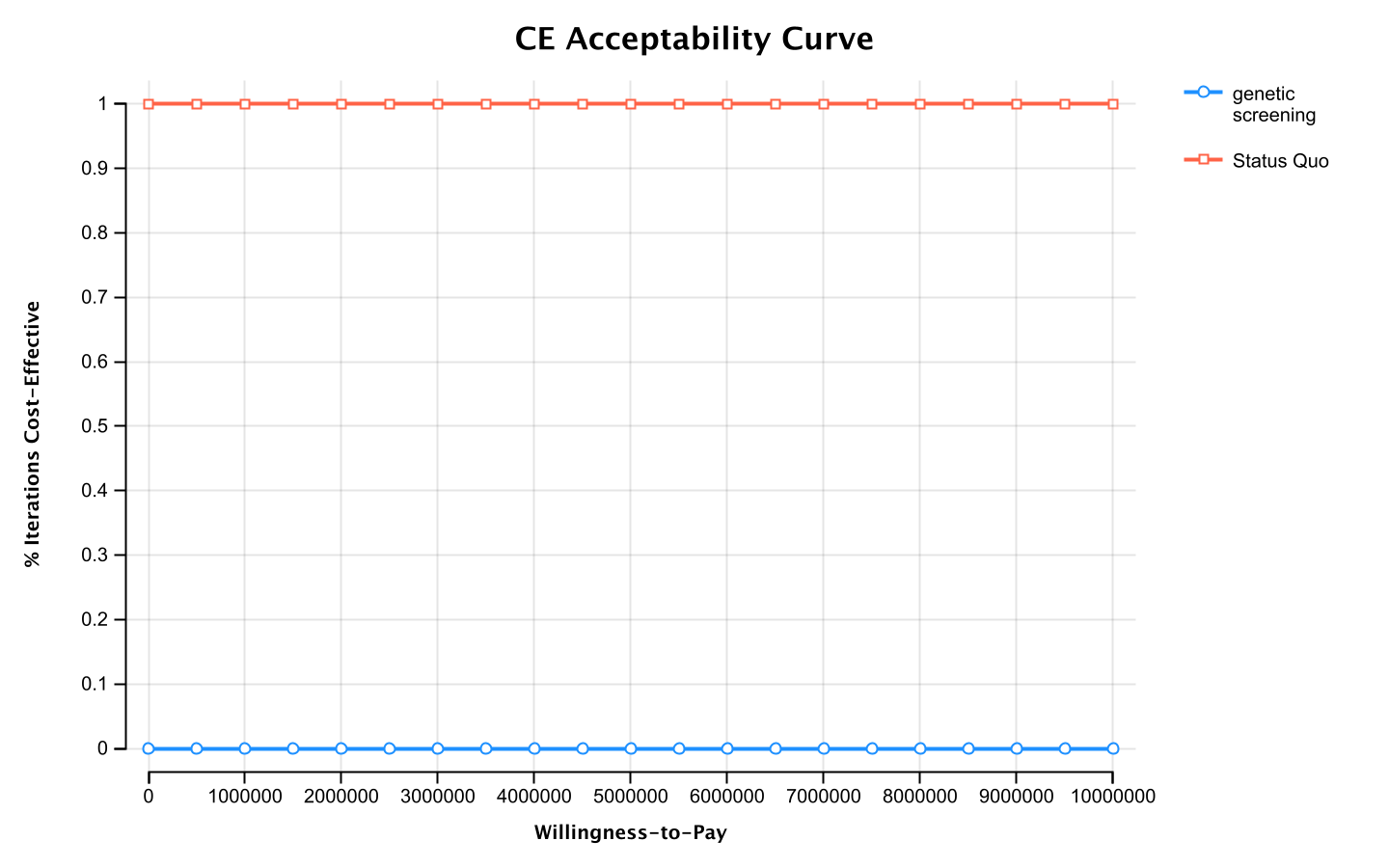


Figure S9-d Model 4 (Health=22.1 Deaf=0)


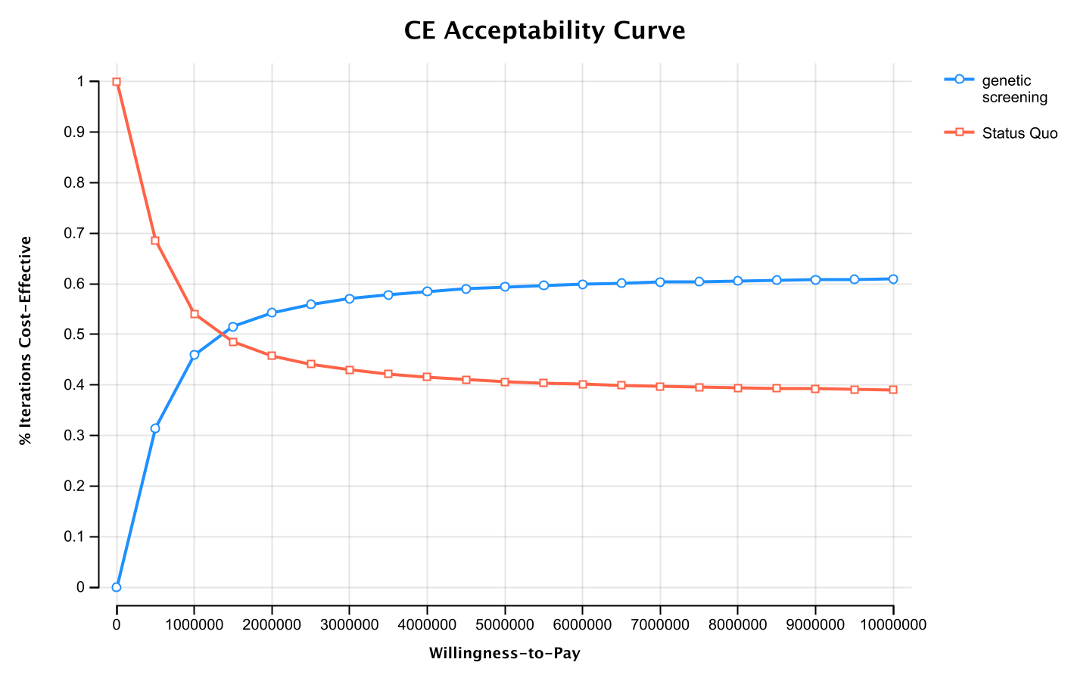


**Table S11 the ICER results of sensitivity analysis in model 1 with all variables**

| Variable Name | Variable Low | Variable Base | Variable High | Impact | ICER Low | ICER High |
| --- | --- | --- | --- | --- | --- | --- |
| pFemNeg | 0.75405 | 0.83783 | 0.92161 | Decrease | -90744.149 | -19910.617 |
| pHusNeg | 0.77 | 0.85556 | 0.94111 | Decrease | -53901.362 | -27215.65 |
| p_MidLowRisk_Nat_Health | 0.675 | 0.75 | 0.825 | Decrease | -46571.103 | -25143.372 |
| cScreen | 51.36 | 64.2 | 77.04 | Decrease | -35580.946 | -29731.089 |
| pFemSingle | 0.14149 | 0.15721 | 0.17293 | Decrease | -35381.069 | -30049.414 |
| cPGT | 9552.24 | 11940.3 | 14328.36 | Decrease | -34598.051 | -30713.985 |
| cAmniocentesis | 2149.28 | 2686.6 | 3223.92 | Decrease | -34403.868 | -30908.167 |
| pCon_PGT | 0.18 | 0.2 | 0.22 | Decrease | -33280.531 | -32026.227 |
| p_Con_Amn_Positive_Birth | 0.1737 | 0.193 | 0.2123 | Decrease | -33227.346 | -32103.697 |
| p_MidRisk_Nat_Health | 0.45 | 0.5 | 0.55 | Decrease | -33222.686 | -32108.356 |
| p_Con_Non_birth | 0.05085 | 0.0565 | 0.06215 | Increase | -32829.511 | -32483.026 |
| pHusDouble | 0.0462 | 0.0513 | 0.0564 | Increase | -32820.813 | -32492.82 |
| cGiveBirth | 520.8 | 651 | 781.2 | Increase | -32782.686 | -32529.349 |
| pFemDouble_Now | 0.02753 | 0.03059 | 0.03365 | Increase | -32777.008 | -32535.917 |
| pFemDouble | 0.0053 | 0.00589 | 0.00648 | Decrease | -32759.934 | -32552.396 |
| pCon_Nat_Birth | 0.05391 | 0.0599 | 0.06589 | Decrease | -32732.819 | -32580.115 |
| cLostIncome | 11.52 | 14.4 | 17.28 | Decrease | -32684.123 | -32627.912 |
| cManuAbortion | 84.48 | 105.6 | 126.72 | Decrease | -32670.972 | -32641.064 |
| p_Amn_Negtive_deaf | 0.0009 | 0.001 | 0.0011 | Decrease | -32663.777 | -32648.262 |
| pFD_HD_Deliver | 0.1737 | 0.193 | 0.2123 | Decrease | -32660.653 | -32651.384 |
| p_PGT_deaf | 0.0009 | 0.001 | 0.0011 | Decrease | -32658.673 | -32653.362 |
| p_Amn_Positive_health | 0.0009 | 0.001 | 0.0011 | Increase | -32656.571 | -32655.465 |
| p_LowRisk_deaf | 0.0009 | 0.001 | 0.0011 | Increase | -32656.018 | -32656.017 |
| cImplement | 16 | 20 | 24 | Increase | -32656.018 | -32656.018 |

**Table S12 the ICER results of sensitivity analysis in model 2 with all variables**

| Variable Name | Variable Low | Variable Base | Variable High | Impact | Low | High |
| --- | --- | --- | --- | --- | --- | --- |
| pCon_PGT | 0.18 | 0.2 | 0.22 | Decrease | 681592.66 | 6113967.43 |
| p_Con_Non_birth | 0.05085 | 0.0565 | 0.06215 | Increase | 692553.406 | 4704712.69 |
| p_MidRisk_Nat_Health | 0.45 | 0.5 | 0.55 | Increase | 739137.299 | 3243567.55 |
| pHusNeg | 0.77 | 0.85556 | 0.94111 | Increase | 1003356.86 | 1987176.56 |
| pFemDouble | 0.0053 | 0.00589 | 0.00648 | Increase | 967368.785 | 1591820.52 |
| pFemDouble_Now | 0.02753 | 0.03059 | 0.03365 | Decrease | 1059712.23 | 1393575.35 |
| cScreen | 51.36 | 64.2 | 77.04 | Increase | 1096093.32 | 1311759.49 |
| cPGT | 9552.24 | 11940.3 | 14328.36 | Increase | 1132329.65 | 1275523.16 |
| cAmniocentesis | 2149.28 | 2686.6 | 3223.92 | Increase | 1139488.54 | 1268364.26 |
| pHusDouble | 0.0462 | 0.0513 | 0.0564 | Decrease | 1149141.18 | 1264177.84 |
| pCon_Nat_Birth | 0.05391 | 0.0599 | 0.06589 | Increase | 1149775.69 | 1263875.18 |
| p_Amn_Negtive_deaf | 0.0009 | 0.001 | 0.0011 | Increase | 1193474.18 | 1214563.32 |
| cGiveBirth | 520.8 | 651 | 781.2 | Decrease | 1199256.53 | 1208596.27 |
| p_PGT_deaf | 0.0009 | 0.001 | 0.0011 | Increase | 1200327.68 | 1207546.76 |
| cLostIncome | 11.52 | 14.4 | 17.28 | Increase | 1202890.25 | 1204962.55 |
| p_Amn_Positive_health | 0.0009 | 0.001 | 0.0011 | Decrease | 1203175.28 | 1204678.47 |
| cManuAbortion | 84.48 | 105.6 | 126.72 | Increase | 1203375.09 | 1204477.71 |
| p_Con_Amn_Positive_Birth | 0.1737 | 0.193 | 0.2123 | Decrease | 1203515.55 | 1204337.77 |
| pFD_HD_Deliver | 0.1737 | 0.193 | 0.2123 | Increase | 1203923.06 | 1203929.74 |
| p_LowRisk_deaf | 0.0009 | 0.001 | 0.0011 | Decrease | 1203925.89 | 1203926.91 |
| cImplement | 16 | 20 | 24 | Increase | 1203926.4 | 1203926.4 |
| pFemSingle | 0.14149 | 0.15721 | 0.17293 | Increase | -1833984.9 | 7429216.91 |
| p_MidLowRisk_Nat_Health | 0.675 | 0.75 | 0.825 | Increase | -1000679.1 | 1203926.4 |
| pFemNeg | 0.75405 | 0.83783 | 0.92161 | Increase | -323642.29 | 1203926.4 |

**Table S13 the ICER results of sensitivity analysis in model 3 with all variables**

| Variable Name | Variable Low | Variable Base | Variable High | Impact | Low | High |
| --- | --- | --- | --- | --- | --- | --- |
| pHusNeg | 0.77 | 0.85556 | 0.94111 | Decrease | -55404.178 | -27974.445 |
| cScreen | 51.36 | 64.2 | 77.04 | Decrease | -36572.974 | -30560.017 |
| cPGT | 9552.24 | 11940.3 | 14328.36 | Decrease | -35562.674 | -31570.317 |
| cAmniocentesis | 2149.28 | 2686.6 | 3223.92 | Decrease | -35363.078 | -31769.913 |
| pCon_PGT | 0.18 | 0.2 | 0.22 | Decrease | -34988.958 | -32194.871 |
| pFemSingle | 0.14149 | 0.15721 | 0.17293 | Increase | -34838.074 | -32512.816 |
| p_Con_Non_birth | 0.05085 | 0.0565 | 0.06215 | Increase | -34463.19 | -32708.86 |
| p_Con_Amn_Positive_Birth | 0.1737 | 0.193 | 0.2123 | Decrease | -34170.752 | -32982.914 |
| cGiveBirth | 520.8 | 651 | 781.2 | Increase | -33696.695 | -33436.295 |
| pHusDouble | 0.0462 | 0.0513 | 0.0564 | Increase | -33695.625 | -33438.313 |
| pFemDouble | 0.0053 | 0.00589 | 0.00648 | Increase | -33685.943 | -33448.306 |
| pCon_Nat_Birth | 0.05391 | 0.0599 | 0.06589 | Decrease | -33603.098 | -33530.23 |
| cLostIncome | 11.52 | 14.4 | 17.28 | Decrease | -33595.384 | -33537.607 |
| cManuAbortion | 84.48 | 105.6 | 126.72 | Decrease | -33581.866 | -33551.124 |
| pFD_HD_Deliver | 0.1737 | 0.193 | 0.2123 | Decrease | -33571.39 | -33561.602 |
| p_LowRisk_deaf | 0.0009 | 0.001 | 0.0011 | Increase | -33566.495 | -33566.495 |
| cImplement | 16 | 20 | 24 | Increase | -33566.495 | -33566.495 |
| p_PGT_deaf | 0.0009 | 0.001 | 0.0011 | Increase | -33566.495 | -33566.495 |
| p_Amn_Positive_health | 0.0009 | 0.001 | 0.0011 | Increase | -33566.495 | -33566.495 |
| p_Amn_Negtive_deaf | 0.0009 | 0.001 | 0.0011 | Increase | -33566.495 | -33566.495 |
| p_MidRisk_Nat_Health | 0.45 | 0.5 | 0.55 | Increase | -33566.495 | -33566.495 |
| p_MidLowRisk_Nat_Health | 0.675 | 0.75 | 0.825 | Increase | -33566.495 | -33566.495 |
| pFemNeg | 0.75405 | 0.83783 | 0.92161 | Increase | -33566.495 | -33566.495 |
| pFemDouble_Now | 0.02753 | 0.03059 | 0.03365 | Increase | -33566.495 | -33566.495 |

**Table S14 the ICER results of sensitivity analysis in model 4 with all variables**

| Variable Name | Variable Low | Variable Base | Variable High | Impact | Low | High |
| --- | --- | --- | --- | --- | --- | --- |
| pFemNeg | 0.754 | 0.83783 | 0.9216 | Decrease | -5726.5125 | -2749.758 |
| pHusNeg | 0.77 | 0.85556 | 0.94111 | Decrease | -5425.8781 | -4057.4184 |
| cFutureIncome_d | 52796.8 | 65996 | 79195.2 | Decrease | -5013.2668 | -3659.5407 |
| eResult_deaf | 18.09 | 20.1 | 22.11 | Increase | -4834.7399 | -3931.1992 |
| pFemSingle | 0.14149 | 0.15721 | 0.17293 | Decrease | -4527.0853 | -4100.6959 |
| cFutMed_d | 8913.92 | 11142.4 | 13370.88 | Increase | -4450.6816 | -4222.1259 |
| p_MidLowRisk_Nat_Health | 0.74267 | 0.75 | 0.75733 | Decrease | -4403.7901 | -4268.6125 |
| cPGT | 9552.24 | 11940.3 | 11940.3 | Decrease | -4336.4038 | -4236.8151 |
| cAmniocentesis | 2149.28 | 2686.6 | 2686.6 | Decrease | -4336.4038 | -4246.7729 |
| p_MidRisk_Nat_Health | 0.45 | 0.5 | 0.55 | Decrease | -4375.8126 | -4296.8568 |
| cFutureIncome_h | 92497.6 | 115622 | 138746.4 | Increase | -4368.569 | -4304.2385 |
| pFemDouble | 0.0276 | 0.00589 | 0.0337 | Decrease | -4690.5279 | -4626.8913 |
| cScreen | 61.36 | 64.2 | 64.2 | Decrease | -4336.4038 | -4303.2279 |
| p_Con_Amn_Positive_Birth | 0.1737 | 0.193 | 0.193 | Decrease | -4336.4038 | -4308.403 |
| eResult_h | 19.8 | 22.1 | 24.2 | Decrease | -4349.1076 | -4322.5749 |
| pHusDouble | 0.0462 | 0.0513 | 0.0564 | Increase | -4345.607 | -4327.2658 |
| pCon_PGT | 0.18 | 0.2 | 0.2 | Decrease | -4336.4038 | -4318.9363 |
| pFemDouble_Now | 0.0275 | 0.03059 | 0.0336 | Increase | -4345.0307 | -4327.9939 |
| cGiveBirth | 520.8 | 651 | 781.2 | Increase | -4342.8994 | -4329.9081 |
| p_Con_Non_birth | 0.5085 | 0.0565 | 0.6285 | Decrease | -4465.7838 | -4456.589 |
| pCon_Nat_Birth | 0.05391 | 0.0599 | 0.0599 | Decrease | -4336.4038 | -4331.7082 |
| cFutMed_h | 5569.12 | 6961.4 | 8353.68 | Decrease | -4338.3404 | -4334.4671 |
| cManuAbortion | 84.48 | 105.6 | 126.72 | Decrease | -4337.1706 | -4335.6369 |
| cLostIncome | 11.52 | 14.4 | 14.5 | Decrease | -4336.4538 | -4334.9625 |
| p_Amn_Negtive_deaf | 0.0009 | 0.001 | 0.0011 | Decrease | -4336.9536 | -4335.8539 |
| pFD_HD_Deliver | 0.1737 | 0.193 | 0.193 | Decrease | -4336.4038 | -4336.1686 |
| p_PGT_deaf | 0.00095 | 0.001 | 0.00104 | Decrease | -4336.479 | -4336.3096 |
| p_Amn_Positive_health | 0.0009 | 0.001 | 0.0011 | Increase | -4336.4429 | -4336.3646 |
| p_LowRisk_deaf | 0.00099 | 0.001 | 0.00101 | Increase | -4336.4038 | -4336.4038 |
| p_Nat_Abor | 0.14185 | 0.15016 | 0.15847 | Increase | -4336.4038 | -4336.4038 |
| p_PGT_Abor | 0.15466 | 0.18019 | 0.21923 | Increase | -4336.4038 | -4336.4038 |
| p_Amn_Abor | 0.13783 | 0.15314 | 0.16845 | Increase | -4336.4038 | -4336.4038 |

Table S15 CHEERS 22022 checklist

| **Topic** | **No.** | **Item** | **Location where item is reported** |
| --- | --- | --- | --- |
| **Title** | | | |
|  | 1 | Identify the study as an economic evaluation and specify the interventions being compared. | Title, Page 1 |
| **Abstract** | | | |
|  | 2 | Provide a structured summary that highlights context, key methods, results, and alternative analyses. | abstract, Pages 1 |
| **Introduction** | | | |
| **Background and objectives** | 3 | Give the context for the study, the study question, and its practical relevance for decision making in policy or practice. | Intro, Pages 3 |
| **Methods** | | | |
| **Health economic analysis plan** | 4 | Indicate whether a health economic analysis plan was developed and where available. | Methods (“Study Design), Page 4. |
| **Study population** | 5 | Describe characteristics of the study population (such as age range, demographics, socioeconomic, or clinical characteristics). | Page 6 |
| **Setting and location** | 6 | Provide relevant contextual information that may influence findings. | Methods (“Study Design), Page 4. |
| **Comparators** | 7 | Describe the interventions or strategies being compared and why chosen. | Methods ("Model generation ") Page.6 |
| **Perspective** | 8 | State the perspective(s) adopted by the study and why chosen. | Page 4 |
| **Time horizon** | 9 | State the time horizon for the study and why appropriate. | N/A (decision tree model instead of Markov model) |
| **Discount rate** | 10 | Report the discount rate(s) and reason chosen. | Page 8 |
| **Selection of outcomes** | 11 | Describe what outcomes were used as the measure(s) of benefit(s) and harm(s). | Page 10 |
| **Measurement of outcomes** | 12 | Describe how outcomes used to capture benefit(s) and harm(s) were measured. | Page 10 |
| **Valuation of outcomes** | 13 | Describe the population and methods used to measure and value outcomes. | Page 10 |
| **Measurement and valuation of resources and costs** | 14 | Describe how costs were valued. | Page 7 |
| **Currency, price date, and conversion** | 15 | Report the dates of the estimated resource quantities and unit costs, plus the currency and year of conversion. | Page 8 |
| **Rationale and description of model** | 16 | If modelling is used, describe in detail and why used. Report if the model is publicly available and where it can be accessed. | Page 7 Figure2 &Figure S2 |
| **Analytics and assumptions** | 17 | Describe any methods for analysing or statistically transforming data, any extrapolation methods, and approaches for validating any model used. | Page 6 |
| **Characterising heterogeneity** | 18 | Describe any methods used for estimating how the results of the study vary for subgroups. | Page 12 |
| **Characterising distributional effects** | 19 | Describe how impacts are distributed across different individuals or adjustments made to reflect priority populations. | Page 13 |
| **Characterising uncertainty** | 20 | Describe methods to characterise any sources of uncertainty in the analysis. | Page 13 |
| **Approach to engagement with patients and others affected by the study** | 21 | Describe any approaches to engage patients or service recipients, the general public, communities, or stakeholders (such as clinicians or payers) in the design of the study. | Page 6 |
| **Results** |  |  |  |
| **Study parameters** | 22 | Report all analytic inputs (such as values, ranges, references) including uncertainty or distributional assumptions. | Page 9 table 1 Table 2 table 3 |
| **Summary of main results** | 23 | Report the mean values for the main categories of costs and outcomes of interest and summarise them in the most appropriate overall measure. | Page 15 Table 4 |
| **Effect of uncertainty** | 24 | Describe how uncertainty about analytic judgments, inputs, or projections affect findings. Report the effect of choice of discount rate and time horizon, if applicable. | Table 3 Figure 3-6 and Table S7-S13 Figure S4-S9 |
| **Effect of engagement with patients and others affected by the study** | 25 | Report on any difference patient/service recipient, general public, community, or stakeholder involvement made to the approach or findings of the study | N/A |
| **Discussion** |  |  |  |
| **Study findings, limitations, generalisability, and current knowledge** | 26 | Report key findings, limitations, ethical or equity considerations not captured, and how these could affect patients, policy, or practice. | Page 18-21 |
| **Other relevant information** |  |  |  |
| **Source of funding** | 27 | Describe how the study was funded and any role of the funder in the identification, design, conduct, and reporting of the analysis | Page 22 |
| **Conflicts of interest** | 28 | Report authors conflicts of interest according to journal or International Committee of Medical Journal Editors requirements. | Page 22 |
